# Phytochrome diversity in green plants and the origin of canonical plant phytochromes

**DOI:** 10.1038/ncomms8852

**Published:** 2015-07-28

**Authors:** Fay-Wei Li, Michael Melkonian, Carl J. Rothfels, Juan Carlos Villarreal, Dennis W. Stevenson, Sean W. Graham, Gane Ka-Shu Wong, Kathleen M. Pryer, Sarah Mathews

**Affiliations:** 1 Department of Biology, Duke University, Durham, North Carolina 27708, USA; 2 Botany Department, Cologne Biocenter, University of Cologne, 50674 Cologne, Germany; 3 University Herbarium and Department of Integrative Biology, University of California, Berkeley, California 94720, USA; 4 Royal Botanic Gardens Edinburgh, Edinburgh EH3 5LR, UK; 5 New York Botanical Garden, Bronx, New York 10458, USA; 6 Department of Botany, University of British Columbia, Vancouver, British Columbia, Canada V6T 1Z4; 7 Department of Biological Sciences, University of Alberta, Edmonton, Alberta, Canada T6G 2E9; 8 Department of Medicine, University of Alberta, Edmonton, Alberta, Canada T6G 2E1; 9 BGI-Shenzhen, Shenzhen 518083, China; 10 Harvard University Herbaria, Cambridge, Massachusetts 02138, USA

## Abstract

Phytochromes are red/far-red photoreceptors that play essential roles in diverse plant morphogenetic and physiological responses to light. Despite their functional significance, phytochrome diversity and evolution across photosynthetic eukaryotes remain poorly understood. Using newly available transcriptomic and genomic data we show that canonical plant phytochromes originated in a common ancestor of streptophytes (charophyte algae and land plants). Phytochromes in charophyte algae are structurally diverse, including canonical and non-canonical forms, whereas in land plants, phytochrome structure is highly conserved. Liverworts, hornworts and *Selaginella* apparently possess a single phytochrome, whereas independent gene duplications occurred within mosses, lycopods, ferns and seed plants, leading to diverse phytochrome families in these clades. Surprisingly, the phytochrome portions of algal and land plant neochromes, a chimera of phytochrome and phototropin, appear to share a common origin. Our results reveal novel phytochrome clades and establish the basis for understanding phytochrome functional evolution in land plants and their algal relatives.

## Abstract (web summary)

Phytochromes are red-light photoreceptors in plants that regulate key life cycle processes, yet their evolutionary origins are not well understood. Using transcriptomic and genomic data, Li *et al.* find that canonical plant phytochromes originated in a common ancestor of land plants and charophyte algae.

## Graphical abstract

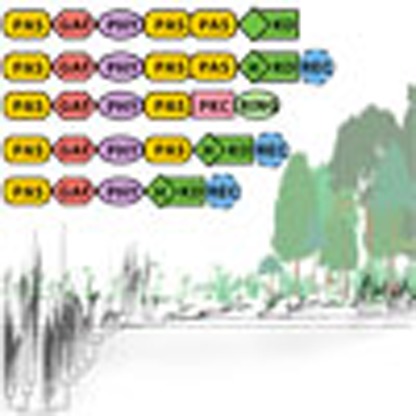

Plants use an array of photoreceptors to measure the quality, quantity and direction of light, in order to respond to ever-changing light environments^1^. Four photoreceptor gene families—the phytochromes, phototropins, Zeitlupes and cryptochromes—along with UVR8, together regulate the majority of developmental and physiological processes mediated by Ultraviolet B and visible light^1^,^2^.

Phytochromes are red/far-red light sensors, particularly prominent for their control of seed germination, seedling photomorphogenesis, shade avoidance, dormancy, circadian rhythm, phototropism and flowering^1^,^3^,^4^. Because of their biological significance, phytochromes have been a major focus in plant research. Phytochrome photochemistry, function and its associated signal transduction mechanisms have been investigated extensively, mostly using the model flowering plant *Arabidopsis thaliana*^1^,^3^,^4^,^5^.

Canonical plant phytochromes comprise an N-terminal photosensory core module (PCM) and a C-terminal regulatory module^3^,^4^. The PCM contains three conserved domains in the linear sequence Per/Arnt/Sim (PAS), cGMP phosphodiesterase/adenylate cyclase/FhlA (GAF) and phytochrome (PHY). It is essential for light reception and photoconversion between reversible conformations that absorb maximally in the red (650–670 nm) or far-red (705–740 nm) regions of the spectrum, referred to as Pr and Pfr, respectively. The C-terminal module consists of a PAS–PAS repeat followed by a histidine kinase-related domain. The histidine kinase-related domain resembles a histidine kinase domain but lacks the conserved histidine phosphorylation site, exhibiting serine/threonine kinase activity instead^6^,^7^.

Plant phytochromes occur as a small nuclear-encoded gene family, and in seed plants they fall into three distinct clades: *PHYA*, *PHYB/E* and *PHYC*^8^. The phylogenetic relationships among these clades are well resolved, allowing for the formulation of functional hypotheses for seed-plant phytochromes based on their orthology with *Arabidopsis* phytochromes^8^. Phytochrome diversity in non-seed plants, however, is very poorly understood, with the limited available data being derived from the *Physcomitrella* (moss) and *Selaginella* (lycophyte) genome projects^9^,^10^, and a few cloning studies^11^,^12^,^13^,^14^,^15^. The lack of a comprehensive phytochrome evolutionary framework for all land plants is an obstacle to understanding the evolution of phytochrome functional diversity, and makes it difficult, for example, to interpret correctly results from comparisons of function in *A. thaliana* and *Physcomitrella patens*.

An especially remarkable plant phytochrome derivative is neochrome, a chimeric photoreceptor combining a phytochrome PCM and a blue light-sensing phototropin^16^. Neochromes have been detected only in zygnemetalean algae, ferns and hornworts^17^,^18^. While it has been shown that the phototropin component of neochromes has two independent origins (one in zygnemetalean algae and the other in hornworts)^18^, the ancestry of the phytochrome portion remains unclear.

In addition to plants, phytochromes are present in prokaryotes, fungi and several protistan and algal lineages^19^,^20^. These phytochromes share with canonical plant phytochromes the PCM domain architecture at the N-terminal, but they differ in their C-terminal regulatory modules. Prokaryotic and fungal phytochromes, for example, lack the PAS–PAS repeat, and have a functional histidine kinase domain with the conserved histidine residue. Recently, Rockwell *et al.*^20^ and Duanmu *et al.*^21^ examined the phytochromes in several algal lineages (brown algae, cryptophytes, glaucophytes and prasinophytes), and discovered that some of them not only exhibit great spectral diversity, but also have novel domain combinations within the C-terminal module. Despite these important findings, phytochromes remain unreported from the majority of algal lineages. Duanmu *et al.*^21^ proposed that the canonical plant phytochrome may have originated among charophyte algae, but they were unable to confirm this.

In this study, we investigated newly available genomic and transcriptomic resources to discover phytochrome homologues outside of seed plants. We examined a total of 300 genomes and transcriptomes from seed plants, ferns, lycophytes, bryophytes, charophytes, chlorophytes and prasinophytes (all in Viridiplantae), and from other plastid-bearing algal lineages, the glaucophytes, cryptophytes, rhodophytes, haptophytes and stramenopiles. We used these data to reconstruct the first detailed phytochrome phylogeny for the eukaryotic branches of the tree of life, and to map all the major gene duplication events and domain architecture transitions onto this evolutionary tree. We uncover new phytochrome lineages and reveal that the canonical plant phytochromes originated in an ancestor of streptophytes (charophyte algae and land plants).

## Results

### Phytochrome phylogenetic reconstructions

We discovered a total of 350 phytochrome homologues in 148 transcriptome assemblies and 12 whole-genome sequences (Supplementary Tables 1 and 2) spanning extant plant and algal diversity. In the remaining 140 assemblies and genome sequences, we detected no phytochrome homologues. We inferred a phytochrome phylogeny from an amino acid matrix that included the sequences we discovered, together with previously published sequences from GenBank. To improve our understanding of phytochrome and neochrome evolution, especially within ferns and bryophytes, we also assembled three nucleotide matrices. The fern and bryophyte matrices included 113 and 97 phytochrome sequences, respectively. The neochrome matrix included 16 neochromes and 95 phytochromes from selected bryophytes and charophytes.

The topologies of our phytochrome gene trees correspond well with published organismal relationships^22^,^23^,^24^,^25^,^26^,^27^,^28^,^29^,^30^,^31^, allowing us to pinpoint the phylogenetic positions of gene duplication events and delineate novel phytochrome clades. Below we report results on phytochrome diversity, phylogenetic structure and domain architecture in the stramenopiles, cryptophytes and Archaeplastida (or ‘Plantae': red algae+glaucophytes+Viridiplantae)^32^.

### Names for phytochrome gene lineages

The high diversity of phytochromes we discovered in charophytes, mosses and ferns—resulting from multiple, independent gene duplications—demanded a sensible system for naming the gene lineages. Within each major organismal group of Archaeplastida (except seed plants, where a system for naming *PHY* has already been well established), we used numerical labels for the phytochrome clades that resulted from major gene duplication events (for example, fern *PHY1-4* and charophyte *PHY1-2*). Subclades resulting from more local duplications were then named alphabetically within clades (for example, Polypodiales *PHY4A-B* and Desmidiales *PHY2A-C*). It should be stressed that this alphanumeric system does not imply orthology across organismal groups; for example fern *PHY1* has a lower degree of relatedness to charophyte *PHY1* than to fern *PHY2*. Charophyte *PHYX1* and *PHYX2* were so named here because they are not canonical plant phytochromes like charophyte *PHY1-2*, and their evolutionary origin is less clear. For phytochromes in glaucophytes, we adopted the term glaucophyte phytochrome sensors (GPS) of Rockwell *et al.*^20^, and for the cryptophyte phytochromes with C-terminal serine/threonine kinase, we followed Duanmu *et al.*^21^ and called them phytochrome eukaryotic kinase hybrids (PEK).

### Stramenopiles and haptophytes

**Figure 1:**
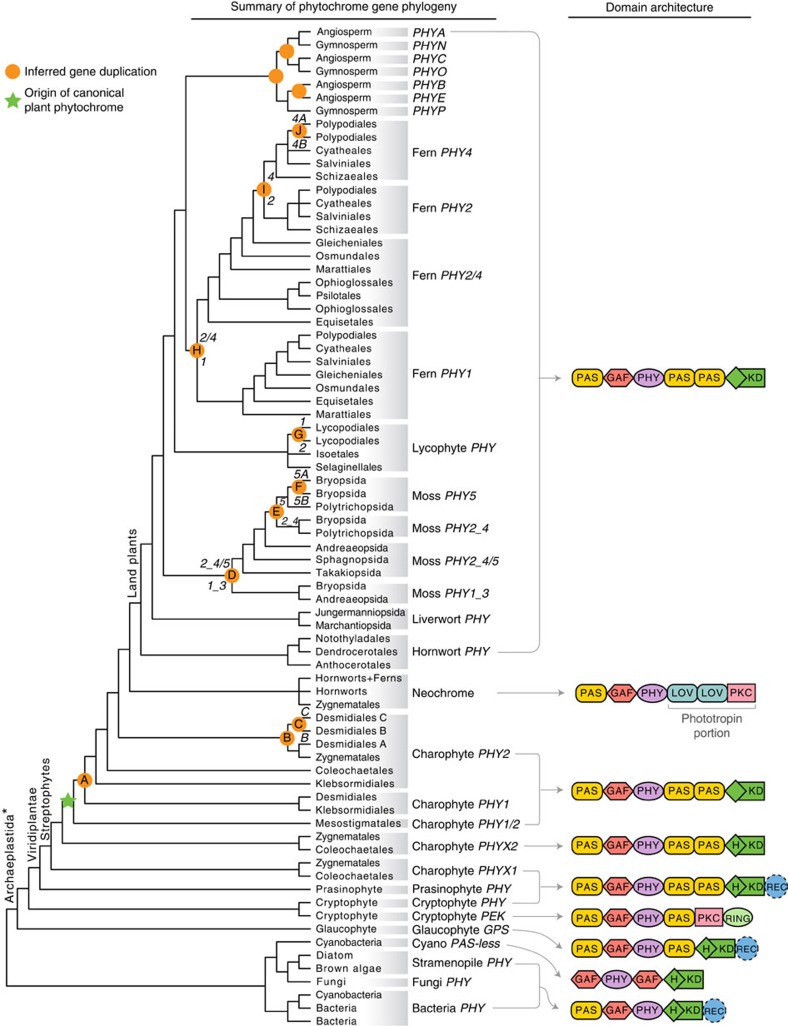
Phylogeny of phytochromes. Terminal clades are collapsed into higher taxonomic units (usually orders or classes) for display purposes. Orange circles indicate inferred gene duplications. Italicized capital letters within each circle correspond to duplication events mentioned in the text, and the numbers/letters adjacent to each orange circle are the names of gene duplicates. Canonical plant phytochromes originated in an ancestor of streptophytes (green star), and some charophyte algae retain non-canonical phytochromes (*PHYX1* and *PHYX2*). Phytochrome domain architectures are shown on the right. Domains that are not always present are indicated by dashed outlines. Domain names: GAF (cGMP phosphodiesterase/adenylate cyclase/FhlA); H/KD (histidine phosphorylation site (H) in the histidine kinase domain (KD)); PAS (Per/Arnt/Sim); PHY (Phytochrome); PKC (Protein Kinase C); REC (Response Regulator); and RING (Really Interesting New Gene). *Traditional Archaeplastida do not include cryptophytes^32^.

Stramenopiles are a large eukaryotic clade that includes brown algae (such as kelps), golden algae and diatoms, the latter being an important component of plankton. Within this group, phytochromes are known so far only from brown algae, some of their viruses and diatoms. Their sequences form a clade that is sister to fungal phytochromes (Fig. 1 and Supplementary Fig. 2). Interestingly, the phytochrome from the brown algal virus EsV-1 (ref. 33) does not group with brown algae phytochromes, but instead is more closely related to those of diatoms. This relationship was not supported in a bootstrapping analysis (Supplementary Fig. 2); it was, however, also obtained by Duanmu *et al.*^21^ (but without support). Additional phytochrome data from stramenopiles will be necessary to clarify the origin of these viral phytochromes. We also examined haptophytes, a predominantly marine lineage of phytoplankton (their relationships with stramenopiles and other protists are unclear^27^,^28^). No phytochrome could be found in the haptophyte transcriptomes.

### Red algae

Red algae are mostly multicellular, marine species that include many coralline reef-building algae. No phytochromes were found in the 28 red algal transcriptomes we examined, nor in the published genomes of *Porphyridium purpureum*, *Chondrus crispus*, *Cyanidioschyzon merolae*, *Galdieria sulphuraria* and *Pyropia yezoensis* (Supplementary Table 1). This result, based on data from all Rhodophyta classes^34^, provides compelling evidence for the absence of phytochromes from red algae (Supplementary Fig. 1).

### Glaucophytes

Glaucophytes are a small clade of freshwater, unicellular algae with unusual plastids referred to as cyanelles, which, unlike plastids in rhodophytes and green plants, retain a peptidoglycan layer^35^. Phytochromes are present in glaucophytes (GPS^20^), and when our tree is rooted on the branch to prokaryote/fungus/stramenopile phytochromes, GPS are resolved as sister to cryptophyte+Viridiplantae phytochromes (Fig. 1 and Supplementary Fig. 2). GPS, in contrast with canonical plant phytochromes, have a single PAS domain in the C-terminal module, and the conserved histidine residue is present in the kinase domain, suggesting it retains histidine kinase activity^21^.

### Cryptophytes

**Figure 2:**
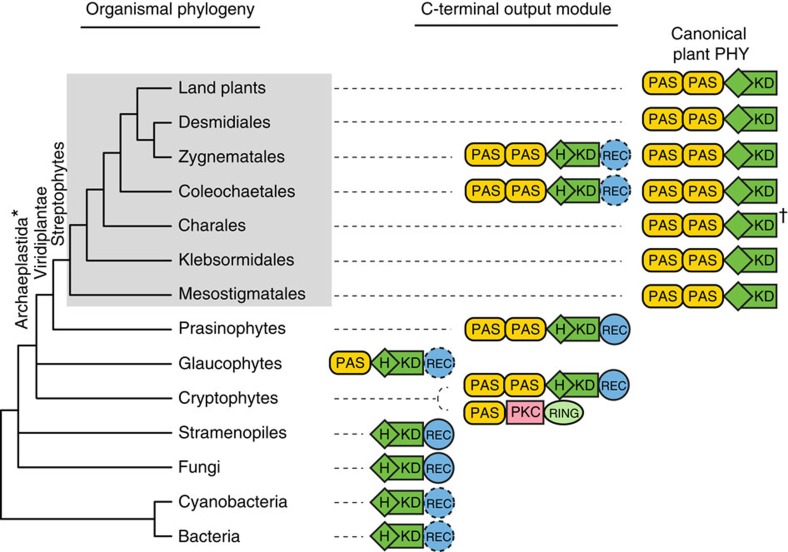
The diversity and evolution of phytochrome C-terminal output module. The tree depicts the organismal phylogeny of all the phytochrome-containing lineages. The domain architecture of the C-terminal regulatory module characteristic of each lineage is indicated on the right connected by dashed lines. The N-terminal photosensory module has a largely conserved domain sequence of PAS–GAF–PHY, and is not drawn here. The substitution of the histidine phosphorylation site (H) in the histidine kinase domain (KD) occurred subsequent to the divergence of prasinophytes. The canonical plant phytochrome is restricted to streptophytes (in grey box); Zygnematales and Coleochaetales also have non-canonical plant phytochromes. Domain names: PAS (Per/Arnt/Sim); PKC (Protein Kinase C); REC (Response Regulator); and RING (Really Interesting New Gene). *Traditional Archaeplastida do not include cryptophytes^32^. †Full-length phytochrome was not available from Charales and its domain structure was inferred.

The phylogenetic position of cryptophytes remains controversial. They were once thought to be related to stramenopiles and haptophytes (belonging to the kingdom Chromalveolata), but some recent phylogenomic studies place them either as nested within, or sister to, Archaeplastida^26^,^27^,^28^. In our analyses, cryptophyte+Viridiplantae phytochromes form a clade that is sister to glaucophyte phytochromes (Fig. 1 and Supplementary Fig. 2). Also, phytochromes from Viridiplantae and from some cryptophytes share the characteristic PAS–PAS repeat in the C terminus (Fig. 2). These cryptophyte phytochromes differ from the canonical phytochromes in their retention of the conserved histidine phosphorylation site in the kinase domain (Figs 1 and 2). Some cryptophyte phytochromes do not have the PAS–PAS repeat in the C terminus, but instead possess a single PAS followed by a serine/threonine kinase domain (‘PKC' in Figs 1 and 2). Despite this variation in the C terminus, the N-terminal photosensory modules of all cryptophyte phytochromes are monophyletic (Fig. 1 and Supplementary Fig. 2).

### Viridiplantae

Viridiplantae comprise two lineages, Chlorophyta and Streptophyta. Chlorophyta include chlorophytes (Trebouxiophyceae+Ulvophyceae+Chlorophyceae+Pedinophyceae) and prasinophytes (Supplementary Fig. 1). Chlorophytes appear to lack phytochromes entirely; we did not find homologues in any of the chlorophyte transcriptomes examined, including 14 Trebouxiophyceae, 21 Ulvophyceae, 59 Chlorophyceae and 2 Pedinophyceae. This result is consistent with available whole-genome sequence data; the genomes of *Chlamydomonas reinhardtii*, *Volvox carteri* and *Chlorella variabilis* (Chlorophyceae) lack phytochromes. Prasinophytes, on the other hand, do have phytochromes. Most of these have a PAS–PAS repeat, a histidine kinase domain, and a response regulator domain at the C terminus^21^. Prasinophyte phytochromes are monophyletic and are the sister group to streptophyte phytochromes (Fig. 1 and Supplementary Fig. 2).

Streptophyta (or streptophytes) are an assemblage of the charophytes (a paraphyletic grade of algae) and the land plants^22^ (Supplementary Fig. 1). We found phytochrome homologues in all land plant clades, as well as in all charophyte lineages: Mesostigmatales (including Chlorokybales), Klebsormidiales, Coleochaetales, Charales, Zygnematales and Desmidiales (Fig. 1 and Supplementary Fig. 1). The Charales phytochromes were not included in our final phylogenetic analyses because the transcriptome contigs (and also the data currently available on GenBank) are too short to be informative about their relationships. All streptophytes have canonical plant phytochromes, including Mesostigmatales, the earliest diverging charophyte lineage (Figs 1 and 2, Supplementary Fig. 1). This result suggests that the origin of the canonical plant phytochrome took place in the ancestor of extant streptophytes.

Within charophyte algae we identified several gene duplication events. We infer one duplication to have occurred after Mesostigmatales diverged (‘A' in Fig. 1), resulting in two clades: one is small and charophyte specific (charophyte *PHY1*), whereas the other is large and includes charophyte *PHY2*, and the land plant phytochromes. Members of the charophyte *PHY1* clade are not common in our algal transcriptomes, and were found only in Desmidiales and in *Entransia* of the early-diverging Klebsormidiales (Supplementary Fig. 2). On the other hand, the charophyte *PHY2* homologue is found consistently across algal transcriptomes. It experienced additional duplications (‘B' and ‘C' in Fig. 1) that resulted in three phytochrome subclades within Desmidiales (Desmidiales *PHY2A-C*). Relationships recovered within each of these phytochrome subclades correspond well to species phylogenies for Desmidiales^25^.

We found that Zygnematales and Coleochaetales (charophytes) also have two non-canonical phytochrome clades (charophyte *PHYX1* and *PHYX2*, Fig. 1). Some *PHYX1* have a response regulator domain at the C terminus, similar to prasinophyte, cryptophyte and glaucophyte phytochromes (Figs 1 and 2). Intriguingly, *PHYX1* lacks all the known conserved cysteine residues (CysA-D^20^) in the PAS–GAF region of the N terminus that bind bilin chromophores, indicating that this protein may not bind a bilin, or that a non-conserved binding site is used.

### Neochromes

**Figure 3:**
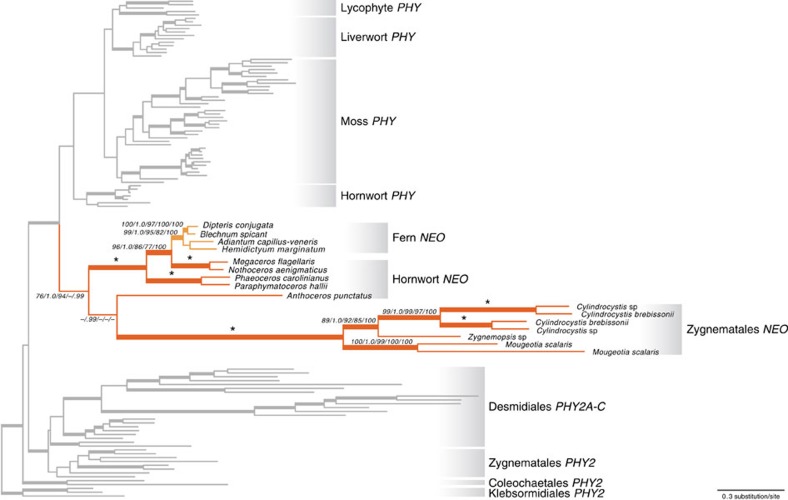
Phylogeny of neochromes and phytochromes. The support values are shown for the neochrome branches only, in the following order: maximum likelihood bootstrap support (BS) from general time reversible (GTR) nucleotide model/Bayesian posterior probabilities (PP) from GTR nucleotide model/aLRT support from codon model/maximum likelihood bootstrap values from Jones-Taylor-Thornton (JTT) amino acid model/Bayesian posterior probabilities from JTT amino acid model. ‘*' Indicates all the support values=100 or 1.0. ‘−' denotes BS<70, aLRT<70, or PP<0.95. Branches are thickened when BS>70, aLRT>70, and PP>0.95.

Our data suggest that the phytochrome module of neochrome had a single origin (Fig. 1 and Supplementary Fig. 2). Published data indicate that the phototropin module of neochromes, in contrast, had independent origins in algae and hornworts^18^, implying two separate fusion events involving phytochromes that shared a common ancestor. To further explore this finding, we analysed the neochrome nucleotide data set (see above) using several nucleotide, codon and amino acid models, and performed a topology test. We consistently recovered the monophyly of the phytochrome module of neochromes, and usually with high support, from analyses using all models (Fig. 3). Although *Anthoceros* (a hornwort) neochrome was resolved as sister to a Zygnematales (algal) neochrome, this relationship was not supported (except in the MrBayes analysis of the nucleotide data set). We then used the Swofford–Olsen–Waddell–Hillis (SOWH) test to compare the topology with all neochromes (the phytochrome module) forming a single clade, against an alternative in which neochromes of Zygnematales were forced to not group with hornworts+ferns. The alternative hypothesis was rejected (*P*<0.00001), and the monophyly of the phytochrome module of neochromes was favoured.

### Bryophytes

**Figure 4:**
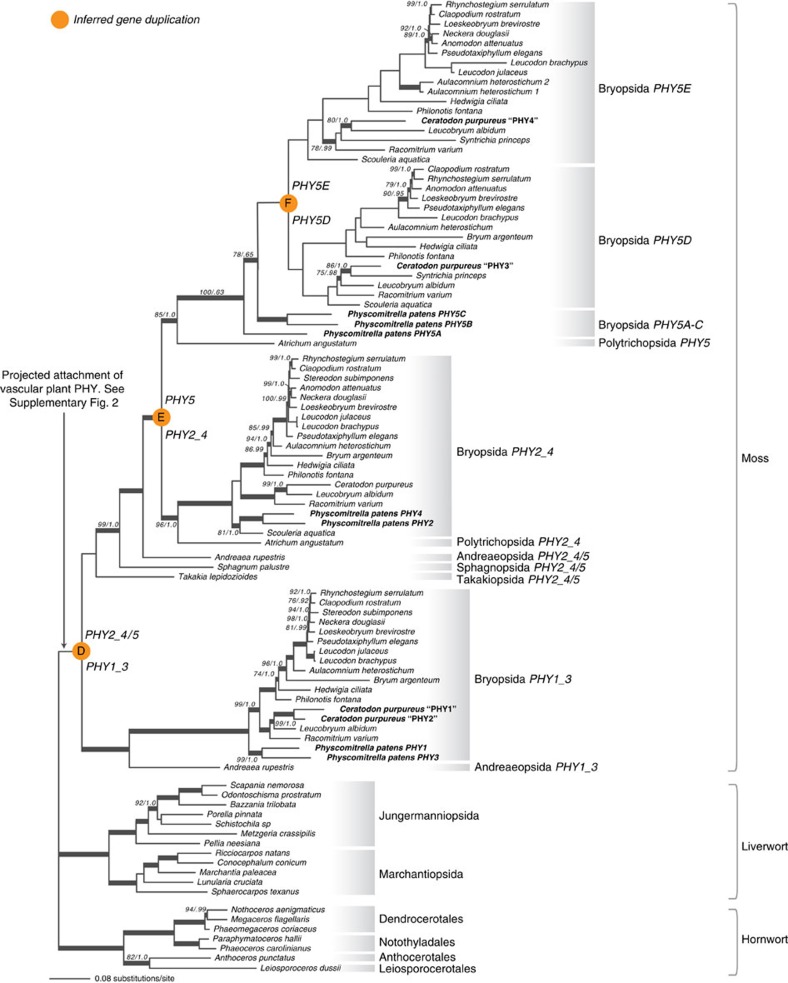
Phylogeny of bryophyte phytochromes. Previously identified phytochromes are in bold font. Support values associated with branches are maximum likelihood bootstrap values (BS)/Bayesian posterior probabilities (PP); these are only displayed (along with thickened branches) if BS>70 and PP>0.95. Thickened branches without numbers are 100/1.0. The position of orange circles indicates inferred gene duplications. Italicized capital letters within each circle correspond to the duplication event mentioned in the text, and the numbers/letters adjacent to each circle indicate the names of the gene duplicates.

Phytochromes from mosses, liverworts and hornworts each form a monophyletic group (Fig. 4). We detected single phytochrome homologues in hornwort and liverwort transcriptomes. The gene phylogenies match the species relationships^30^,^31^, consistent with the presence of single orthologous genes in these taxa. Indeed, a single phytochrome has been identified via cloning methods in the liverwort, *Marchantia paleacea* var*. diptera*^15^. We also searched the low-coverage draft genome of the hornwort *Anthoceros punctatus* (20X; Li *et al.*^18^) and found only one phytochrome. To further evaluate gene copy number, we hybridized the *A. punctatus* genomic DNA with phytochrome RNA probes, and used Illumina MiSeq to sequence the captured DNA fragments. The same phytochrome contig (and only that contig) was recovered, suggesting that this hornwort does not harbour additional, divergent phytochrome copies.

**Figure 5:**
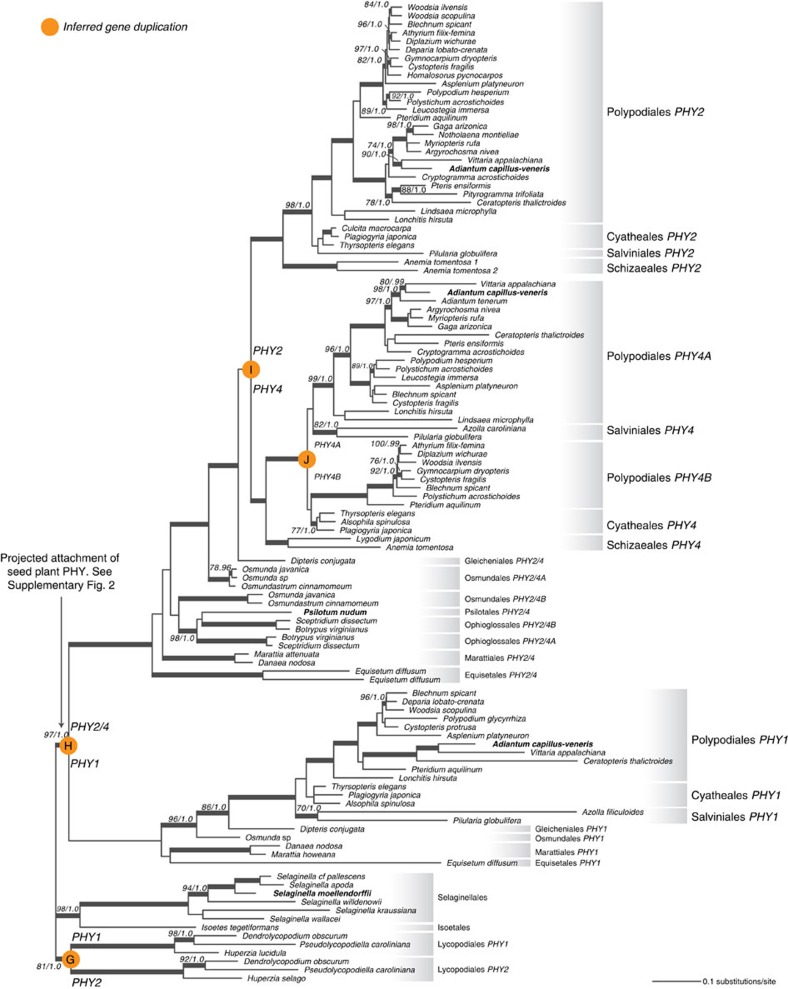
Phylogeny of fern and lycophyte phytochromes. Previously identified phytochromes are shown in bold. Support values associated with branches are maximum likelihood bootstrap values (BS)/Bayesian posterior probabilities (PP); these are only displayed (along with thickened branches) if BS>70 and PP>0.95. Thickened branches without numbers are 100/1.0. The position of orange circles estimates the origin of inferred gene duplications. Italicized capital letters within each circle correspond to the duplication event mentioned in the text, and the numbers/letters adjacent to each circle indicate the names of the gene duplicates.

In contrast, phytochromes in mosses are diverse, with at least four distinct clades resulting from three gene duplications (Fig. 4). The phylogeny reveals those moss phytochromes that are orthologous to the previously named *P. patens* phytochromes, *PpPHY1–5*. The *Physcomitrella* phytochromes and their orthologs form the following clades: moss *PHY1_3* (including *PpPHY1* and *PpPHY3*), moss *PHY2_4* (including *PpPHY2* and *PpPHY4*), and moss *PHY5* (including *PpPHY5A–C*). An ancient duplication (‘D' in Fig. 4) gave rise to moss *PHY1*_*3* and moss *PHY2_4*+*PHY5* clades. The timing of this duplication is dependent on the phylogenetic position of the *Takakia* phytochrome, resolved here as sister to the moss *PHY2_4*+*PHY5* clade, but without support (Fig. 4). Because *Takakia* (Takakiopsida) represents the earliest diverging lineage in the moss species phylogeny^36^, the first phytochrome duplication probably predates the origin of all extant mosses. In the moss *PHY2_4*+*PHY5* clade, another duplication (‘E' in Fig. 5) occurred following the split of *Andreaea* (Andreaeopsida) but before *Atrichum* (Polytrichopsida) diverged, separating moss *PHY2_4* and *PHY5*. The moss *PHY5* clade had an additional duplication (‘F' in Fig. 4), probably after *Physcomitrella* diverged, that resulted in moss *PHY5D* and *PHY5E* subclades.

Our results show that the phytochrome copies previously cloned from *Ceratodon purpureus*, which were named *CpPHY1–4* (ref. 37), have the following relationships with the moss phytochromes: *CpPHY1* and *CpPHY2* are each others closest relatives, and are members of the moss *PHY1_3* lineage; *CpPHY3* and *CpPHY4* are members of the moss *PHY5* lineage (Fig. 4). These results suggest that the four known *C. purpureus* phytochromes—‘*CpPHY1*', ‘*CpPHY2*', ‘*CpPHY3*' and ‘*CpPHY4*' (Fig. 4) should be renamed to *CpPHY1_3A*, *CpPHY1_3B, CpPHY5D* and *CpPHY5E*, respectively, and that the novel *C. purpureus* phytochrome discovered here should be designated as *CpPHY2_4*.

### Lycophytes

Lycophyte phytochromes are resolved as monophyletic and are sister to the fern plus seed-plant phytochromes (Figs 1 and 5, Supplementary Fig. 2). *Selaginella* and *Isoetes* (Isoetopsida) each have a single phytochrome, with the exception of *Selaginella mollendorffii*, where two nearly identical phytochromes are apparent in the whole-genome sequence data. Their high degree of similarity suggests that they might be products of a species-specific gene duplication. In contrast, Lycopodiales have two distinct phytochrome clades that we name Lycopodiales *PHY1* and Lycopodiales *PHY2*. Because all the Lycopodiales lineages^38^ are represented in each phytochrome clade, we infer that the duplication of Lycopodiales *PHY1/2* (‘G' in Fig. 5) predates the common ancestor of all extant Lycopodiales.

### Ferns

Fern phytochromes form a clade that is sister to the seed plant phytochromes (Figs 1 and 5, Supplementary Fig. 2). Within ferns we uncovered four phytochrome clades that we designate fern *PHY1*, *PHY2*, *PHY4A* and *PHY4B*. The name *PHY3* was used previously to denote the chimeric photoreceptor that is now recognized as neochrome^17^,^18^. The deep evolutionary split between the fern *PHY1* and *PHY2/4* clades predates the most recent ancestor of extant ferns (‘H' in Fig. 5). Fern *PHY2* and *PHY4* probably separated after Gleicheniales diverged (‘I' in Fig. 5), and the earliest diverging fern lineages (that is, Gleicheniales, Osmundales, Psilotales, Ophioglossales, Marattiales and Equisetales) have the pre-duplicated *PHY2/4* copy. It should be noted that our broad-scale amino acid data set resolved a slightly different topology, placing Gleicheniales *PHY2/4* closer to *PHY4* (Supplementary Fig. 2). However, the amino acid data set included fewer sequences from ferns, which could reduce phylogenetic accuracy^39^. It is likely that that the phylogeny (Fig. 5) inferred from rigorous analyses of nucleotide data more accurately reflects gene relationships.

We found that Ophioglossales and Osmundales each have two *PHY2/4* copies, which likely arose from independent gene duplications (Fig. 5). The duplication of Ophioglossales *PHY2/4A* and *PHY2/4B* occurred either at the ancestor of Ophioglossales or of Ophioglossales+Psilotales, but the history of *PHY2/4* in Osmundales is unclear. The Osmundales *PHY2/4A* and *PHY2/4B* were not resolved as monophyletic, and the phylogenetic position of Osmundales *PHY2/4B* is incongruent with published fern species relationships^23^.

After the split of fern *PHY2* and *PHY4*, *PHY4* duplicated again, giving rise to fern *PHY4A* and *PHY4B* (‘J' in Fig. 5), and both are found in Polypodiales. We cannot precisely determine the timing of this duplication event because the relationships among Polypodiales *PHY4A-B*, Cyatheales *PHY4* and Salviniales *PHY4* are resolved without support. Interestingly, *PHY4A* was previously known only from *Adiantum capillus-veneris* (as *AcPHY4*). Its first intron incorporated an inserted Ty3/gypsy retrotransposon and the downstream exon sequence was unknown^12^. We found full-length *PHY4A* transcripts in a wide range of Polypodiales, suggesting that *PHY4A* likely is functional in most other species, if not in *A. capillus-veneris*. *PHY4B* is a novel phytochrome clade that has not been documented before; it is not common in the fern transcriptomes we examined.

### Seed plants

Seed plant phytochromes cluster into three clades (Supplementary Fig. 2) corresponding to *PHYA*, *PHYB/E* and *PHYC*, in accordance with previous studies^8^. Organismal relationships within the gene subclades largely are consistent with those inferred in phylogenetic studies of angiosperms^40^. Notably, however, support for the monophyly of gymnosperms was low. We found two divergent transcripts of *PHYE* in Ranunculales, represented by *Aquilegia* (Ranunculaceae; from whole-genome data) and *Capnoides* (Papaveraceae; from transcriptome data) (Supplementary Fig. 2), suggesting that a gene duplication event occurred deep in Ranunculales; however, more extensive sampling in Ranunculales is needed to resolve the timing of this duplication.

## Discussion

Our phylogenetic results refute previous hypotheses suggesting that plants acquired phytochrome from cyanobacteria via endosymbiotic gene transfer^41^,^42^, because streptophyte and cyanobacterial phytochromes are not closest relatives in our phytochrome trees (Fig. 1 and Supplementary Fig. 2), a result also recently obtained by Duanmu *et al.*^21^. Instead, plant phytochromes evolved from a precursor shared with other Archaeplastida. We clearly placed the origin of canonical plant phytochromes in a common ancestor of extant streptophytes (Figs 1 and 2). Our data, moreover, show that the origin of this structure required multiple steps. The gain of the internal PAS–PAS repeat took place first, in the ancestor of Viridiplantae, or of Viridplantae+cryptophytes (Fig. 2). As noted above, the position of cryptophytes is uncertain, and its inclusion in Archaeplastida is not strongly supported in published studies^26^,^27^,^28^. The topology of our phytochrome trees is consistent with a sister-group relationship between Viridiplantae and cryptophytes, but the topology also could result from endosymbiotic or horizontal gene transfer. The loss of the histidine phosphorylation site in the histidine kinase domain—hence the attainment of the canonical form—occurred later, in the ancestor of streptophytes, and seems to have been accompanied by a permanent dissociation with the response regulator at the C-terminal end (Fig. 2). Some streptophytes have additional, non-canonical phytochromes. Charophyte *PHYX1* and *PHYX2*, both found in Zygnematales and Coleochaetales, have the conserved histidine residue, and some *PHYX1* also have a response regulator domain (Figs 1 and 2). The fact that charophyte *PHYX1* and *PHYX2* were not found in all streptophytes implies that some orthologs may have been missed in our transcriptomic and genomic scans, and/or a scenario in which duplications occurred early in streptophytes and were followed by multiple losses.

Our findings highlight the different evolutionary modes of the phytochrome N- and C-terminal modules. The N-terminal photosensory module is deeply conserved across eukaryotes and prokaryotes, and the linear domain sequence of PAS–GAF–PHY is found in the majority of known phytochromes (Fig. 1). In contrast, the evolution of the C-terminal regulatory module has been much more dynamic (Fig. 2). For example, the C-terminal PAS may be absent, may occur singly, or may occur as a tandem repeat (Fig. 3). Serine/threonine kinase or tyrosine kinase domains have also been independently recruited into the regulatory module in the cryptophyte and *C. purpureus* (moss) phytochromes^43^ (Fig. 2). The successful linkage of the phytochrome photosensory module with a variety of C-terminal modules has promoted phytochrome functional diversity. Certainly the most compelling example is that of the neochromes. It combines phytochrome and phototropin modules into a single protein to process blue and red/far-red light signals in the control of phototropism^44^. Neochrome was first discovered in ferns^16^ and postulated to be a driver of the modern fern radiation under low light, angiosperm-dominated forest canopies^45^,^46^,^47^. Suetsugu *et al.*^17^ later discovered a similar phytochrome–phototropin chimera in *Mougeotia scalaris* (zygnematalean alga), and proposed that neochrome had independently evolved twice. A recent study identified yet another neochrome from hornworts, and demonstrated that ferns acquired their neochromes from hornworts via horizontal gene transfer^18^. By placing the phototropin portion of neochrome into a broad phylogeny of phototropins, Li *et al.*^18^ also showed that phototropin modules of neochromes had two separate origins, once in hornworts and once in zygnematalean algae. In contrast, the phytochrome portion of neochrome has had a different evolutionary history, with Zygnematales, hornworts and ferns forming a single monophyletic group (Fig. 3 and Supplementary Fig. 2). This result is robust, and is supported by most of the analyses and by a topology test. Our results thus suggest that neochromes originated via two separate fusion events, involving two distinct sources of phototropin but the same phytochrome progenitor. This is a fascinating extension of the capacity and propensity of the phytochrome photosensory module to be linked with functionally distinct downstream domains.

The major clades of land plants differ markedly with respect to phytochrome gene diversity. It appears that phytochromes are single copy in most liverworts, hornworts and Isoetopsida (Isoetaceae and Selaginellaceae), whereas they have independently diversified in Lycopodiales, mosses, ferns and seed plants (Fig. 1). In ferns, a pattern of early gene duplication followed by gene losses could explain the phylogenetic positions of two Osmundales *PHY2/4*, which are incongruent with known species relationships in ferns (Fig. 5). Interestingly, we observed a relationship between phytochrome copy number and species richness. For instance, the polypod ferns (Polypodiales), which account for 90% of extant fern diversity^47^, have four phytochrome copies, whereas other species-poor fern lineages have only two or three (Fig. 5). Likewise, moss species belonging to the hyper-diverse Bryopsida—containing 95% of extant moss diversity—have experienced the highest number of phytochrome duplications compared with other bryophyte lineages (Fig. 4). It is possible that the evolution of phytochrome structural and functional diversity enhanced the ability of polypod ferns and Bryopsida mosses to adapt to diverse light environments. Indeed, seed plants, ferns and mosses each have at least one phytochrome duplicate that convergently evolved or retained the role of mediating high-irradiance responses^48^,^49^,^50^,^51^, a trait likely to be important for surviving under deep canopy shade^52^ (see below). This ‘phytochrome-driven species diversification' hypothesis, however, needs rigorous testing by phylogenetic comparative methods and functional studies in non-seed plants that identify the genetic bases of phytochrome functions.

The independent phytochrome diversification events in seed plants, ferns, mosses and Lycopodiales have significant implications for phytochrome functional studies. Moss phytochromes, for example, are more closely related to each other than to any of the seed-plant phytochromes (and the same is true, of course, for phytochromes from ferns and those from Lycopodiales). Seed-plant phytochromes have undergone significant differentiation into two major types. One is represented by phyA of *A. thaliana*, which is the primary mediator of red-light responses in deep shade and beneath the soil surface. It degrades rapidly in light, mediates very-low fluence and high-irradiance responses, and depends on protein partners FHY1 (far-red elongated hypocoytl 1) and FHL (FHY1 like) for nuclear translocation. The other is represented by phyB-E of *A. thaliana*, which are the primary mediators of red-light responses in open habitats. They have a longer half-life than phyA in light, mediate low-fluence responses, and in the case of phyB, nuclear translocation does not require FHY1 or FHL^4^,^5^. A similar partitioning of function has been documented in some fern and moss phytochrome duplicates^49^,^53^,^54^, demonstrating a case of convergent differentiation following independent gene duplications. In future studies, it would be of particular interest to infer the ancestral properties of land plant phytochrome: Did it have a short or long half-life? What kinds of physiological responses did it mediate? How was nuclear translocation executed? Studies of liverwort, hornwort and *Selaginella* phytochromes, which exist as a single-copy gene, could serve as ‘baseline models' for understanding the genetic basis of phytochrome functional diversification.

Recent functional studies on a small but varied set of algal phytochromes revealed a surprising degree of spectral diversity, which might reflect adaptations to a range of marine and aquatic environments^20^,^21^. For example, photoreversible phytochromes in prasinophyte algae include orange/far-red receptors as well as red/far-red receptors, and in algae outside of Viridiplantae, there also are blue/far-red and red/blue receptors^21^. This sharply contrasts with the very limited spectral diversity in canonical plant phytochromes, all of which are red/far-red receptors as far as is known. The novel algal phytochrome clades we detected are a potential treasure trove for discovering the steps involved during the transition from a spectrally diverse set of reversible photoreceptors to a set that is centred on the red to far-red region of the spectrum, and for the characterization of new biochemical variants. Some of these may have implications for understanding the role of phytochrome evolution in the recolonization of marine and freshwater environments by terrestrial plants.

In summary, our study has revealed that the diversity of Viridiplantae phytochromes is far greater than was realized, and points to exciting opportunities to link this structural diversity with function and ecology.

## Methods

### Mining transcriptomes and genomes for phytochrome homologues

The transcriptomes and genomes sampled in this study are listed in Supplementary Table 1. We used the Python pipeline BlueDevil following Li *et al.*^18^ to mine transcriptomes. To search the whole-genome data, we used BLASTp implemented in Phytozome^55^ or individual genome project portals (Supplementary Table 1). The protein domain composition of each of the phytochrome sequences was determined by querying the NCBI Conserved Domain Database^56^.

### Sequence alignment

In addition to the phytochrome homologues mined from transcriptomes and genomes, we gathered selected Genbank accessions and a sequence cloned from *Marattia howeana* (voucher: S.W.Graham and S. Mathews 15, deposited in NSW; primers: 110f –5′GTNACNGCNTAYYTNCARCGNATG3′, 788r – 5′GTMACATCTTGRSCMACAAARCAYAC3′).

We assembled four sequence data sets, one was translated into an amino acid alignment and the others were analysed as nucleotide matrices. The amino acid data set included the majority of the sequences (423 sequences in total; Supplementary Fig. 2). The sequences were initially aligned using MUSCLE^57^, followed by manual curation of the alignment based on known domain boundaries and protein structures. We did not include regions with uncertain or no homology; these include all response regulator (REC), really interesting new gene (RING), Light-Oxygen-Voltage sensor (LOV) and PKC domains, as well as the single PAS domain in GPS. Unalignable regions were also excluded and the final alignment included 1,106 amino acid sites. The nucleotide data sets were assembled to provide higher phylogenetic resolution within fern+lycophyte phytochromes (113 sequences; Fig. 5), bryophyte phytochromes (97 sequences; Fig. 4), and neochromes (111 sequences; Fig. 3). Sequences were aligned as amino acids and then back-translated to nucleotides, and the alignment was refined by manual editing. The fern+lycophyte, and bryophyte phytochrome alignments contained 3,366 and 3,429 nucleotide sites, respectively. The neochrome alignment included only the N-terminal photosenory module (PAS–GAF–PHY domains; 1,920 nucleotide sites). All alignments are available from Dryad (http://dx.doi.org/10.5061/dryad.5rs50).

### Phylogenetic reconstruction

For the broad-scale amino acid alignment, JTT+I+G was selected as the best-fitting empirical model by ProTest 3 under Akaike Information Criterion^58^. We used Garli v2.0 (ref. 59) to find the maximum likelihood tree, with ten independent runs and genthreshfortopoterm set to 100,000. The starting tree for Garli came from a RAxML v8.1.11 (ref. 60) run. To obtain bootstrap branch support values, RAxML was run with 1,000 replicates using JTT+G.

For the nucleotide alignments, we used PartitionFinder v1.1.1 (ref. 61) to infer the best partitioning schemes and substitution models, under Akaike Information Criterion. Maximum likelihood tree searching and bootstrapping (1,000 replicates) were done in RAxML. Bayesian inference was carried out in MrBayes v3.2.3 (ref. 62), with two independent Markov chain Monte Carlo (MCMC) runs and four chains each. We unlinked the substitution parameters and set the rate prior to vary among partitions. The MCMC output was inspected using Tracer^63^ to ensure convergence and mixing (effective sample sizes all >200); 25% of the total generations were discarded as burn-in before analyzing the posterior distribution.

Additional analyses were applied to the neochrome data set. First, we used CodonPhyML v1.0 (ref. 64) to infer the tree topology and to assess support (SH-like aLRT branch support), using a codon substitution model. Four categories of non-synonymous/synonymous substitution rate ratios were drawn from a discrete gamma distribution, and codon frequencies were estimated from the nucleotide frequencies at each codon position (F3 × 4). Second, we translated the nucleotides into amino acids, and carried out maximum likelihood tree searching and bootstrapping (in RAxML), as well as Bayesian inference (in MrBayes) under the JTT+I+G model. Finally, we used the SOWH test, implemented in SOWHAT^65^, to investigate whether the inferred tree topology (phytochrome portion of neochrome forming a clade) is significantly better than the alternative topology (neochrome not monophyletic). In SOWHAT, we used the default stopping criterion and applied a topological constraint forcing land plant and zygnematalean neochrome to be non-monophyletic.

To derive the organismal relationships shown in Fig. 2 and Supplementary Fig. 1, we merged the topologies from three recent phylogenetic studies. The relationships within streptophytes and chlorophyte algae were from Wickett *et al.*^22^ and Marin^29^, respectively. For the broader kingdom-level relationship, we referenced the topology of Grant and Katz^28^.

### Confirming gene copy number by target enrichment

We used a target enrichment strategy to test whether hornworts have a single phytochrome locus. In this approach, specific RNA probes are hybridized to genomic DNA to enrich the representation of particular gene fragments. Target enrichment has several advantages over the traditional Southern blotting approach. In particular, it uses thousands of different hybridization probes (rather than just a few), and the end products are not DNA bands, but actual sequence data.

We designed a total of 7,502 120-mer RNA probes to target phytochrome, phototropin and neochrome genes, with a special focus on those of hornworts and ferns (probe sequences available from Dryad http://dx.doi.org/10.5061/dryad.5rs50). The probes overlap every 60 bp (a 2X tiling strategy), and were synthesized and biotin-labeled by Mycroarray. Genomic DNA of the hornwort *A. punctatus* was extracted using a modified hexadecyl trimethyl-ammonium bromide (CTAB) protocol, and sheared by Covaris with fragments peaking at 300 bp. Library preparation for Illumina sequencing was done using a KAPA Biosystem kit, in combination with NEBNext Multiplex Oligos. To enrich for potentially divergent homologues, we used the touchdown procedure of Li *et al.*^66^, in which the genomic DNA library and the probes were hybridized at 65 °C for 11 h followed by 60 °C (11 h), 55 °C (11 h) and 50 °C (11 h). The hybridized DNA fragments were captured by streptavidin beads and washed following the protocol of Mycroarray. The final product was pooled with nine other libraries in equimolar and sequenced on Illumina MiSeq (250 bp paired end). To process the reads, we used Scythe v0.994 (ref. 67) to remove the adapter sequences, and used Sickle v1.33 (ref. 68) to trim low-quality bases. The resulting reads were then assembled by SOAPdenovo2 (ref. 69), and the phytochrome contig was identified by BLASTn^70^. The raw reads were deposited in NCBI SRA (SRP055877).

### Data availability

All relevant data present in this publication can be accessed at http://dx.doi.org/10.5061/dryad.5rs50.

## Additional information

**Accession codes:** The reads from target enrichment generated in this study have been deposited in the NCBI Sequence Read Archive under the accession code SRP055877. The phytochrome DNA sequences generated in this study have been deposited in the GenBank database under accession codes KP872700, KT003814 to KT003816, KT013280 to KT013293 and KT071819 to KT072044.

**How to cite this article:** Li, F.-W. *et al.* Phytochrome diversity in green plants and the origin of canonical plant phytochromes. *Nat. Commun.* 6:7852 doi: 10.1038/ncomms8852 (2015).

## Supplementary Material

Supplementary Information Supplementary Figures 1-2, Supplementary Tables 1-2 and Supplementary References
