# Supplementary material for: Phytochrome diversity in green plants and the origin of canonical plant phytochromes
Source: Nat Commun. 2015 Jul 28;6:7852. doi: 10.1038/ncomms8852 (PMC4525182; doi:10.1038/ncomms8852)
Supplement: Supplementary Information — Supplementary Figures 1-2, Supplementary Tables 1-2 and Supplementary References [file ncomms8852-s1.pdf]

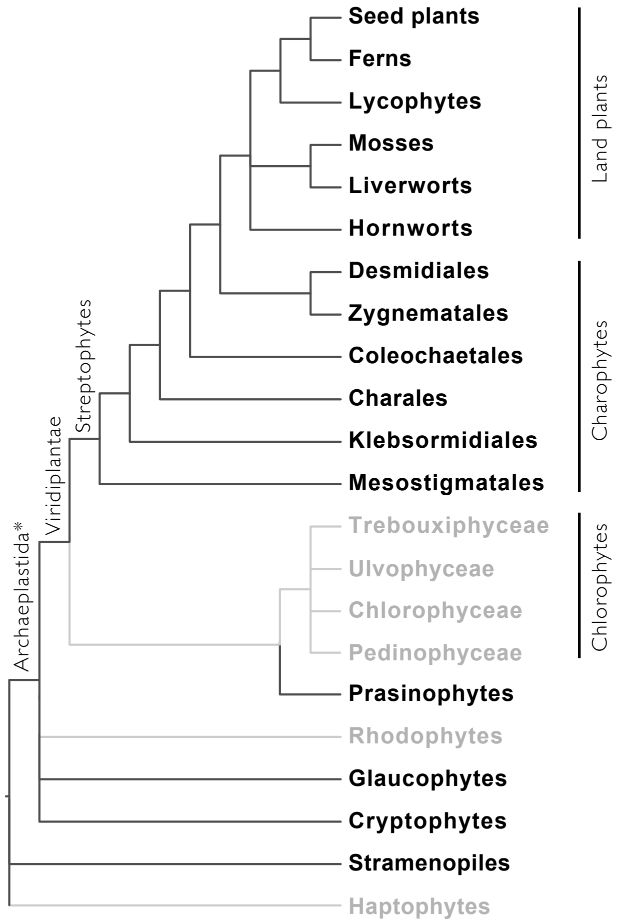

Supplementary Figure 1. The organismal lineages screened for phytochrome homologs. Phytochromes were not detected in lineages indicated in grey. *Traditional Archaeplastida does not include cryptophytes.

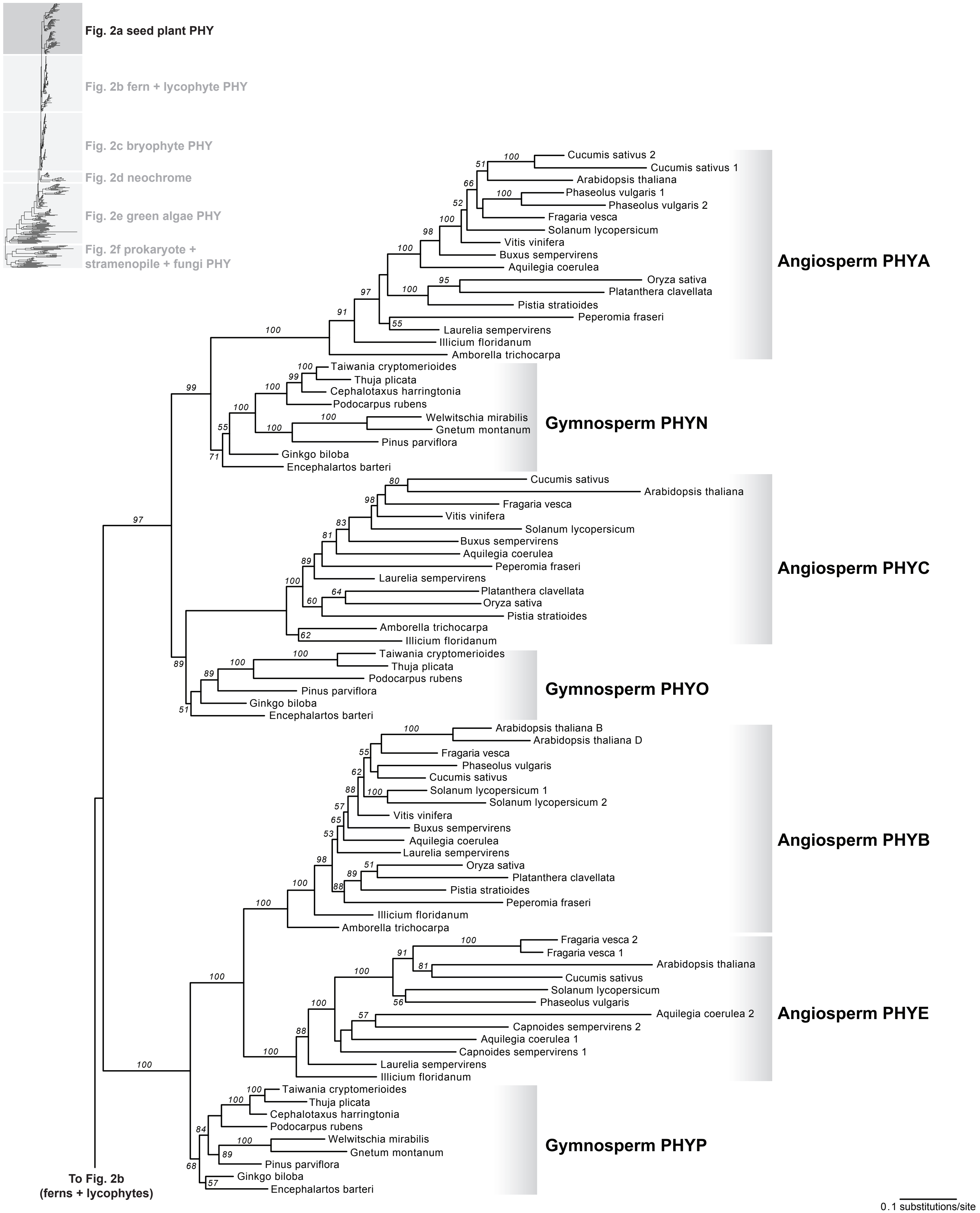

Supplementary Figure 2. The phylogeny of phytochromes reconstructed from 423 protein sequences. Topology derived from the best maximum likelihood tree. The values associated with branches are maximum likelihood bootstrap support. For better visualization, the complete tree is broken down into six subtrees (a-f) and scaled differently. The inset at the top-left corner shows the map to each subtree. **(a)** The seed plant phytochrome subtree. Phylogeny continues in Supplementary Fig. 2b.

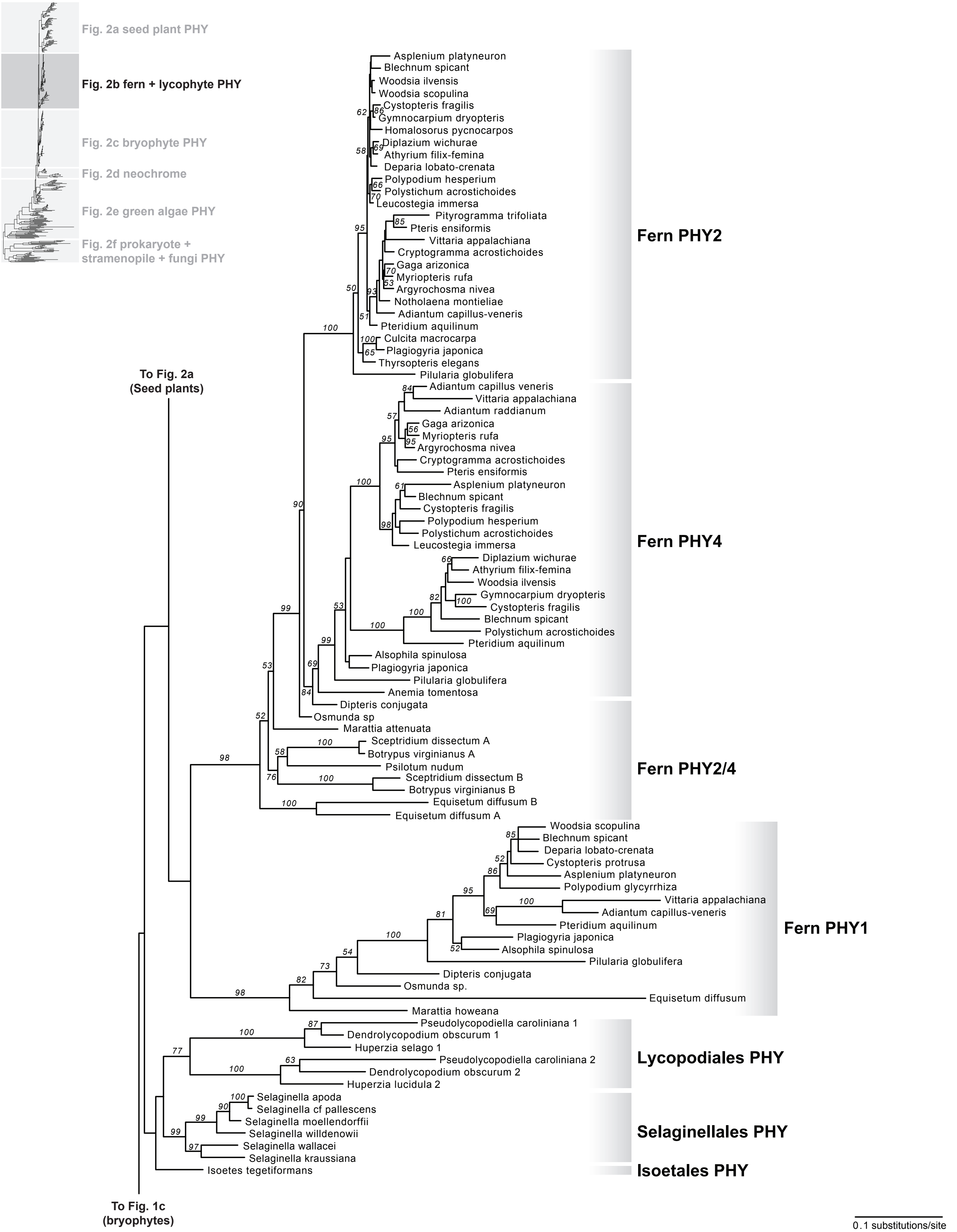

Supplementary Figure 2. (b) The fern and lycophyte phytochrome subtree. Phylogeny continues in Supplementary Fig. 2a,c. See Fig. 5 for a more extensively sampled and higher resolution phylogeny that was reconstructed from nucleotide dataset.

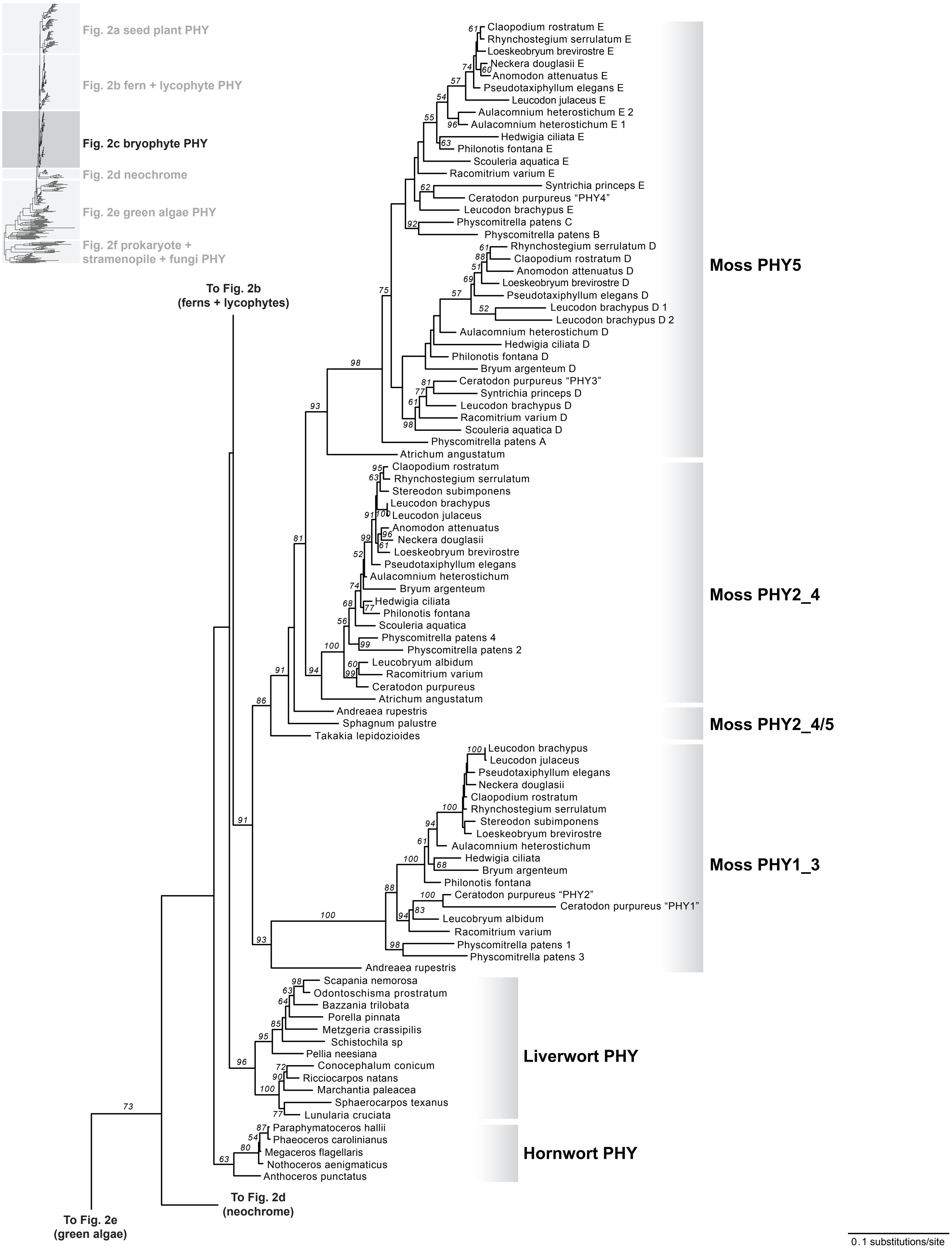

Supplementary Figure 2. (c) The bryophyte phytochrome subtree. Phylogeny continues in Supplementary Fig. 2b,d,e. See Fig. 4 for a more extensively sampled and higher resolution phylogeny that was reconstructed from nucleotide dataset.

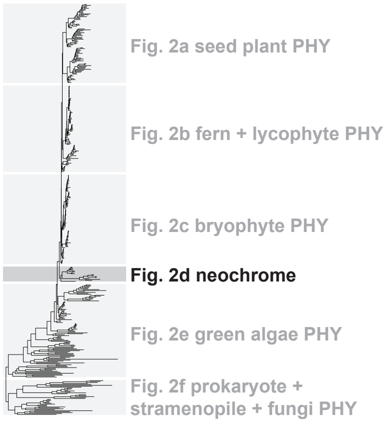

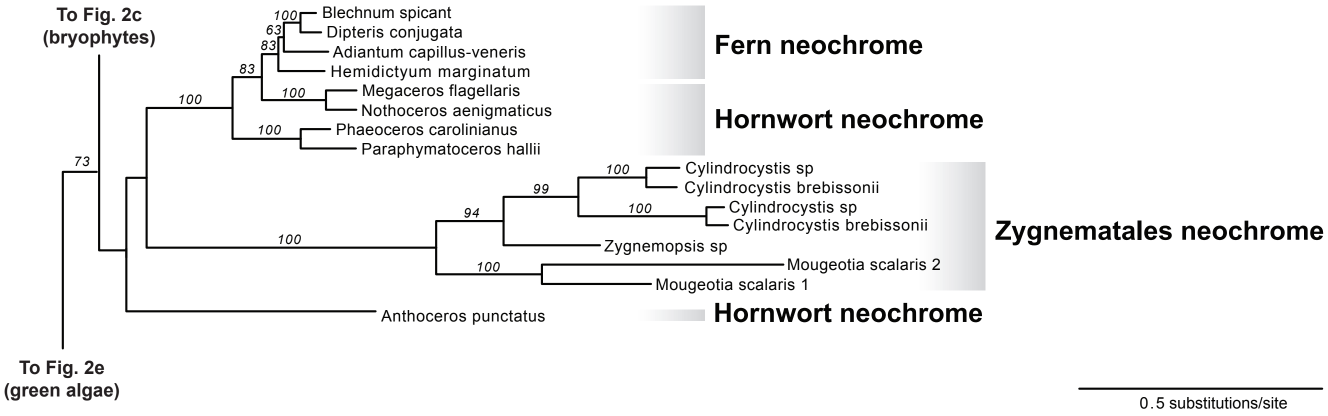

Supplementary Figure 2. (d) The neochrome subtree. Phylogeny continues in Supplementary Fig. 2c,e. See Fig. 3 for a more extensively sampled and higher resolution phylogeny.

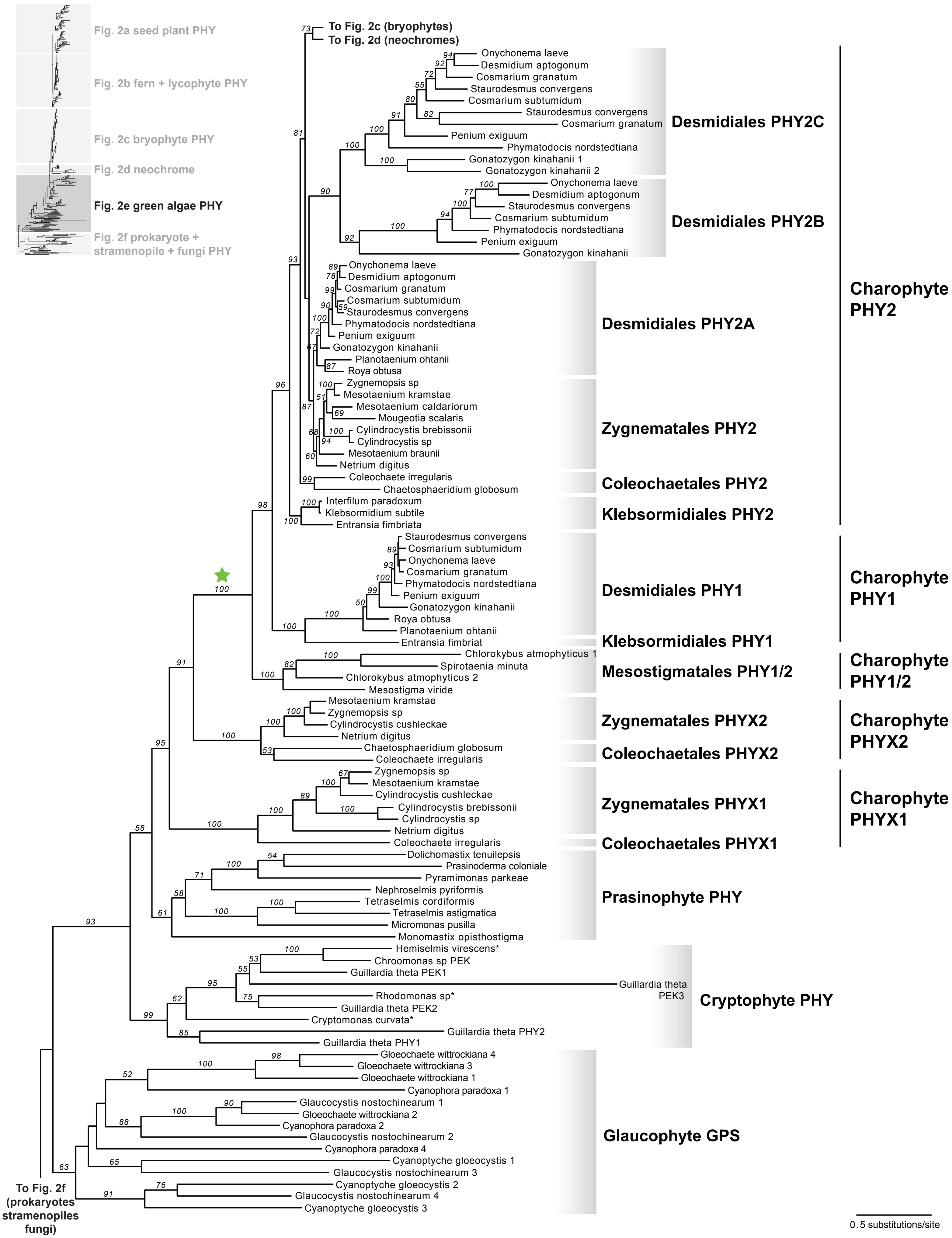

Supplementary Figure 2. (e) The green algae phytochrome subtree. Green star marks the origin of canonical plant phytochrome. Phylogeny continues in Supplementary Fig. 2c,d,f. "*" marks the cryptophyte phytochromes that have incomplete C-terminal end, so that they cannot be determined as PEK or not.

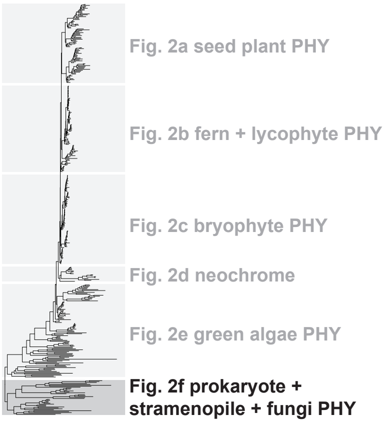

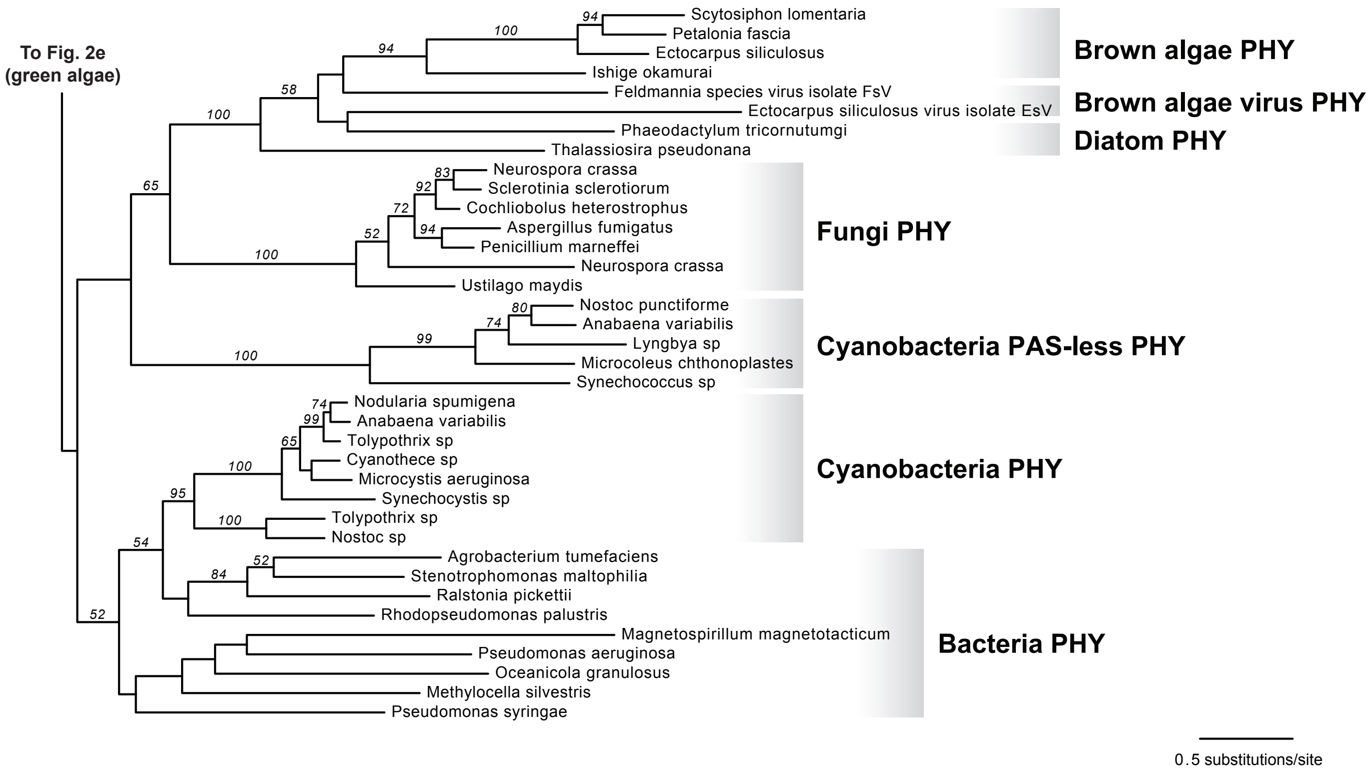

Supplementary Figure 2. (f) The prokaryote + stramenopile + fungi phytochrome subtree. Phylogeny continues in Supplementary Fig. 2e.

Supplementary Table 1. List of transcriptomes and genomes screened for phytochrome homologs. "-": not applicable.

Taxa	Source	1KP[3] identififer
**Angiosperm**		
Amborella trichocarpa	Genome, Amborella Genome Project[1]	-
Aquilegia coerulea	Genome, Phytozome[2]	-
Arabidopsis thaliana	Genome, Phytozome	-
Buxus sempervirens	Transcriptome, 1KP[3]	IWMW
Capnoides sempervirens	Transcriptome, 1KP	AUGV
Cucumis sativus	Genome, Phytozome	-
Fragaria vesca	Genome, Phytozome	-
Illicium floridanum	Transcriptome, 1KP	VZCI
Laurelia sempervirens	Transcriptome, 1KP	WAIL
Oryza sativa	Genome, Phytozome	-
Peperomia fraseri	Transcriptome, 1KP	XSZI
Phaseolus vulgaris	Genome, Phytozome	-
Pistia stratioides	Transcriptome, 1KP	MFIN
Platanthera clavellata	Transcriptome, 1KP	MTHW
Solanum lycopersicum	Genome, Phytozome	-
Vitis vinifera	Genome, Phytozome	-
**Gymnosperm**		
Cephalotaxus harringtonia	Transcriptome, 1KP	WYAJ
Encephalartos barteri	Transcriptome, 1KP	GNQG
Ginkgo biloba	Transcriptome, 1KP	SGTW
Gnetum montanum	Transcriptome, 1KP	GTHK
Pinus parviflora	Transcriptome, 1KP	IIOL
Podocarpus rubens	Transcriptome, 1KP	XLGK
Taiwania cryptomerioides	Transcriptome, 1KP	QSNJ
Thuja plicata	Transcriptome, 1KP	VFYZ
Welwitschia mirabilis	Transcriptome, 1KP	TOXE
**Fern**		
Adiantum tenerum	Transcriptome, 1KP	BMJR
Anemia tomentosa	Transcriptome, 1KP	CQPW
Argyrochosma nivea	Transcriptome, 1KP	XDDT
Asplenium platyneuron	Transcriptome, 1KP	KJZG
Athyrium filix-femina	Transcriptome, 1KP	AFPO
Azolla caroliniana	Transcriptome, 1KP	CVEG
Azolla filiculoides	Transcriptome, Brouwer et al.[4]	-
Blechnum spicant	Transcriptome, 1KP	VITX
Botrypus virginianus	Transcriptome, 1KP	BEGM
Ceratopteris thalictroides	Transcriptome, 1KP	PIVW
Gaga arizonica	Transcriptome, 1KP	DCDT

Cryptogramma acrostichoides	Transcriptome, 1KP	WQML
Culcita macrocarpa	Transcriptome, 1KP	PNZO
Alsophila spinulosa	Transcriptome, 1KP	GANB
Cystopteris fragilis	Transcriptome, 1KP	LHLE
Cystopteris protrusa	Transcriptome, 1KP	YOWV
Danaea nodosa	Transcriptome, 1KP	DFHO
Deparia lobato-crenata	Transcriptome, 1KP	FCHS
Diplazium wichurae	Transcriptome, 1KP	UFJN
Dipteris conjugata	Transcriptome, 1KP	MEKP
Equisetum diffusum	Transcriptome, 1KP	CAPN
Gymnocarpium dryopteris	Transcriptome, 1KP	HEGQ
Homalosorus pycnocarpos	Transcriptome, 1KP	OCZL
Leucostegia immersa	Transcriptome, 1KP	WGTU
Lindsaea microphylla	Transcriptome, 1KP	YIXP
Lonchitis hirsuta	Transcriptome, 1KP	VVRN
Lygodium japonicum	Transcriptome, 1KP	PBUU
Marattia attenuata	Transcriptome, 1KP	UGNK
Myriopteris rufa	Transcriptome, 1KP	GSXD
Notholaena montieliae	Transcriptome, 1KP	YCKE
Osmunda javanica	Transcriptome, 1KP	VIBO
Osmunda sp	Transcriptome, 1KP	UOMY
Osmundastrum cinnamomeum	Transcriptome, 1KP	RFMZ
Pilularia globulifera	Transcriptome, 1KP	KIIX
Pityrogramma trifoliata	Transcriptome, 1KP	UJTT
Plagiogyria japonica	Transcriptome, 1KP	UWOD
Polypodium glycyrrhiza	Transcriptome, 1KP	CJNT
Polypodium hesperium	Transcriptome, 1KP	GYFU
Polystichum acrostichoides	Transcriptome, 1KP	FQGQ
Pteridium aquilinum	Transcriptome, Der et al.[5]	-
Pteris ensiformis	Transcriptome, 1KP	FLTD
Sceptridium dissectum	Transcriptome, 1KP	EEAQ
Thyrsopteris elegans	Transcriptome, 1KP	EWXK
Vittaria appalachiana	Transcriptome, 1KP	NDUV
Woodsia ilvensis	Transcriptome, 1KP	YQEC
Woodsia scopulina	Transcriptome, 1KP	YJJY
**Isoetopsida**		
Selaginella cf pallescens	Transcriptome, 1KP	ABIJ
Selaginella moellendorffii	Genome, Phytozome	-
Selaginella willdenowii	Transcriptome, 1KP	KJYC
Selaginella kraussiana	Transcriptome, 1KP	ZFGK
Selaginella wallacei	Transcriptome, 1KP	JKAA
Selaginella apoda	Transcriptome, 1KP	LGDQ

Isoetes tegetiformans	Transcriptome, 1KP	PKOX
**Lycopodiales**		
Dendrolycopodium obscurum	Transcriptome, 1KP	XNXF
Huperzia selago	Transcriptome, 1KP	GTUO
Huperzia lucidula	Transcriptome, 1KP	GKAG
Pseudolycopodiella caroliniana	Transcriptome, 1KP	UPMJ
**Moss**		
Andreaea rupestris	Transcriptome, 1KP	WOGB
Anomodon attenuatus	Transcriptome, 1KP	QMWB
Claopodium rostratum	Transcriptome, 1KP	VBMM
Atrichum angustatum	Transcriptome, 1KP	ZTHV
Aulacomnium heterostichum	Transcriptome, 1KP	WNGH
Bryum argenteum	Transcriptome, 1KP	JMXW
Ceratodon purpureus	Transcriptome, 1KP	FFPD
Hedwigia ciliata	Transcriptome, 1KP	YWNF
Stereodon subimponens	Transcriptome, 1KP	LNSF
Leucobryum albidum	Transcriptome, 1KP	VMXJ
Leucodon brachypus	Transcriptome, 1KP	ZACW
Neckera douglasii	Transcriptome, 1KP	TMAJ
Philonotis fontana	Transcriptome, 1KP	ORKS
Physcomitrella patens	Genome, Phytozome	-
Pseudotaxiphyllum elegans	Transcriptome, 1KP	QKQO
Racomitrium varium	Transcriptome, 1KP	RDOO
Rhynchostegium serrulatum	Transcriptome, 1KP	JADL
Loeskeobryum brevirostre	Transcriptome, 1KP	WSPM
Leucodon julaceus	Transcriptome, 1KP	IGUH
Scouleria aquatica	Transcriptome, 1KP	BPSG
Sphagnum palustre	Transcriptome, 1KP	RCBT
Syntrichia princeps	Transcriptome, 1KP	GRKU
Takakia lepidozioides	Transcriptome, 1KP	SKQD
**Liverwort**		
Scapania nemorosa	Transcriptome, 1KP	IRBN
Porella pinnata	Transcriptome, 1KP	UUHD
Schistochila sp	Transcriptome, 1KP	LGOW
Metzgeria crassipilis	Transcriptome, 1KP	NRWZ
Pellia neesiana	Transcriptome, 1KP	JHFI
Odontoschisma prostratum	Transcriptome, 1KP	YBQN
Conocephalum conicum	Transcriptome, 1KP	ILBQ
Lunularia cruciata	Transcriptome, 1KP	TXVB
Sphaerocarpos texanus	Transcriptome, 1KP	HERT
Ricciocarpos natans	Transcriptome, 1KP	WJLO
Bazzania trilobata	Transcriptome, 1KP	WZYK

**Hornwort**		
Nothoceros aenigmaticus	Transcriptome, 1KP	DXOU
Megaceros flagellaris	Transcriptome, 1KP	UCRN
Paraphymatoceros hallii	Transcriptome, 1KP	FAJB
Phaeoceros carolinianus	Transcriptome, 1KP	WCZB
Anthoceros punctatus	Genome, Li et al.[6]	-
Phaeomegaceros coriaceus	Transcriptome, 1KP	AKXB
Leiosporoceros dussii	Transcriptome, 1KP	ANON
**Desmidiales**		
Cosmarium granatum	Transcriptome, 1KP	MNNM
Cosmarium subtumidum	Transcriptome, 1KP	WDGV
Desmidium aptogonum	Transcriptome, 1KP	DFDS
Gonatozygon kinahanii	Transcriptome, 1KP	KEYW
Onychonema laeve	Transcriptome, 1KP	GGWH
Penium exiguum	Transcriptome, 1KP	YSQT
Phymatodocis nordstedtiana	Transcriptome, 1KP	RPQV
Planotaenium ohtanii	Transcriptome, 1KP	SNOX
Roya obtusa	Transcriptome, 1KP	XRTZ
Staurodesmus convergens	Transcriptome, 1KP	WCQU
**Zygnematales**		
Cylindrocystis brebissonii	Transcriptome, 1KP	YOXI
Cylindrocystis cushleckae	Transcriptome, 1KP	JOJQ
Cylindrocystis sp	Transcriptome, 1KP	VAZE
Mesotaenium braunii	Transcriptome, 1KP	WSJO
Mesotaenium kramstae	Transcriptome, 1KP	NBYP
Netrium digitus	Transcriptome, 1KP	FFGR
Zygnemopsis sp	Transcriptome, 1KP	MFZO
**Coleochaetales**		
Coleochaete irregularis	Transcriptome, 1KP	QPDY
Chaetosphaeridium globosum	Transcriptome, 1KP	DRGY
**Klebsormidiales**		
Interfilum paradoxum	Transcriptome, 1KP	FPCO
Klebsormidium subtile	Transcriptome, 1KP	FQLP
Entransia fimbriata	Transcriptome, 1KP	BFIK
**Mesostigmatales**		
Chlorokybus atmophyticus	Transcriptome, 1KP	AZZW
Mesostigma viride	Transcriptome, 1KP	KYIO
Spirotaenia minuta	Transcriptome, 1KP	NNHQ
**Charales**		
Chara vulgaris	Transcriptome, 1KP	MWXT
**Trebouxiophyceae**		
Botryococcus braunii	Transcriptome, 1KP	ETGN

Botryococcus terribilis	Transcriptome, 1KP	QYXY
Coccomyxa pringsheimii	Transcriptome, 1KP	GXBM
Eremosphaera viridis	Transcriptome, 1KP	MNCB
Geminella sp.	Transcriptome, 1KP	PFUD
Leptosira obovata	Transcriptome, 1KP	ZNUM
Microthamnion kuetzigianum	Transcriptome, 1KP	DXNY
Nannochloris atomus	Transcriptome, 1KP	MFYC
Neochloris oleoabundans	Transcriptome, 1KP	EEJO
Parachlorella kessleri	Transcriptome, 1KP	AKCR
Prasiola crispa	Transcriptome, 1KP	WCLV
Prototheca wickerhamii	Transcriptome, 1KP	BILC
Stichococcus bacillaris	Transcriptome, 1KP	WXRI
Trebouxia arboricola	Transcriptome, 1KP	NKXU
**Ulvophyceae**		
Acrosiphonia sp.	Transcriptome, 1KP	JIWJ
Blastophysa cf. rhizopus	Transcriptome, 1KP	VHIJ
Bolbocoleon piliferum	Transcriptome, 1KP	LSHT
Bryopsis plumosa	Transcriptome, 1KP	JTIG
Cephaleuros virescens	Transcriptome, 1KP	YDCQ
Cladophora glomerata	Transcriptome, 1KP	VBLH
Codium fragile	Transcriptome, 1KP	GYBH
Desmochloris halophila	Transcriptome, 1KP	KSFK
Entocladia endozoica	Transcriptome, 1KP	OQON
Halochlorococcum marinum	Transcriptome, 1KP	ALZF
Helicodictyon planctonicum	Transcriptome, 1KP	AJAU
Ignatius tetrasporus	Transcriptome, 1KP	KADG
Ochlochaete sp.	Transcriptome, 1KP	CQQP
Oltmannsiellopsis viridis	Transcriptome, 1KP	PZBH
Oltmannsiellopsis viridis	Transcriptome, 1KP	QJYX
Percursaria percursa	Transcriptome, 1KP	OAEZ
Planophila laetevirens	Transcriptome, 1KP	CBNG
Planophila sp.	Transcriptome, 1KP	LETF
Pirula salina	Transcriptome, 1KP	NQYP
Pseudoneochloris marina	Transcriptome, 1KP	GJIY
Trentepohlia annulata	Transcriptome, 1KP	NATT
**Chlorophyceae**		
Ankistrodesmus sp.	Transcriptome, 1KP	OTQG
Aphanochaete repens	Transcriptome, 1KP	IJMT
Asteromonas gracilis	Transcriptome, 1KP	NTLE
Botryosphaerella sudetica	Transcriptome, 1KP	VJDZ
Brachiomonas submarina	Transcriptome, 1KP	GUBD
Carteria crucifera	Transcriptome, 1KP	VIAU

Carteria obtusa	Transcriptome, 1KP	RUIF
Chaetopeltis orbicularis	Transcriptome, 1KP	BAZF
Chlamydomonas reinhardtii	Genome, Phytozome	-
Chlamydomonas bilatus	Transcriptome, 1KP	MULF
Chlamydomonas cribrum	Transcriptome, 1KP	BCYF
Chlamydomonas moewusii	Transcriptome, 1KP	JRGZ
Chlamydomonas noctigama	Transcriptome, 1KP	VALZ
Chlamydomonas sp.	Transcriptome, 1KP	TSBQ
Chlamydomonas sp.	Transcriptome, 1KP	AOUJ
Chlorella variabilis	Genome, Blanc et al.[7]	-
Chloromonas oogama	Transcriptome, 1KP	IHOI
Chloromonas perforata	Transcriptome, 1KP	QRTH
Chloromonas reticulata	Transcriptome, 1KP	LBRP
Chloromonas rosae	Transcriptome, 1KP	AJUW
Chloromonas subdivisa	Transcriptome, 1KP	GFUR
Chloromonas tughillensis	Transcriptome, 1KP	UTRE
Cylindrocapsa geminella	Transcriptome, 1KP	DZPJ
Dunaliella salina	Transcriptome, 1KP	RHVC
Dunaliella tertiolecta	Transcriptome, 1KP	ZDIZ
Eudorina elegans	Transcriptome, 1KP	RNAT
Fritschiella tuberosa	Transcriptome, 1KP	VFIV
Golenkinia longispicula	Transcriptome, 1KP	BZSH
Gonium pectorale	Transcriptome, 1KP	KUJU
Haematococcus pluvialis	Transcriptome, 1KP	ODXI
Haematococcus pluvialis	Transcriptome, 1KP	AGIO
Hafniomonas reticulata	Transcriptome, 1KP	FXHG
Heterochlamydomonas inaequalis	Transcriptome, 1KP	IRYH
Lobochlamys segnis	Transcriptome, 1KP	OFUE
Lobomonas rostrata	Transcriptome, 1KP	JKKI
Microspora cf. tumidula	Transcriptome, 1KP	FOYQ
Neodesmus pupukensis	Transcriptome, 1KP	MWAN
Neochlorosarcina sp.	Transcriptome, 1KP	USIX
Oedogonium cardiacum	Transcriptome, 1KP	DVYE
Oedogonium foveolatum	Transcriptome, 1KP	SDPC
Oogamochlamys gigantea	Transcriptome, 1KP	XDLL
Pandorina morum	Transcriptome, 1KP	RYJX
Pediastrum duplex	Transcriptome, 1KP	XKWQ
Pediastrum duplex	Transcriptome, 1KP	XTON
Phacotus lenticularis	Transcriptome, 1KP	ZIVZ
Pleurastrum insigne	Transcriptome, 1KP	PRIQ
Pteromonas angulosa	Transcriptome, 1KP	LNIL
Pteromonas sp.	Transcriptome, 1KP	ACRY

Scenedesmus dimorphus	Transcriptome, 1KP	PZIF
Spermatozopsis exsultans	Transcriptome, 1KP	MXDS
Spermatozopsis similis	Transcriptome, 1KP	ENAU
Stephanosphaera pluvialis	Transcriptome, 1KP	ZLQE
Stigeoclonium helveticum	Transcriptome, 1KP	JMUI
Uronema sp.	Transcriptome, 1KP	ISGT
Uronema belkae	Transcriptome, 1KP	RAWF
Vitreochlamys sp.	Transcriptome, 1KP	QWRA
Volvox carteri	Genome, Phytozome	-
Volvox aureus	Transcriptome, 1KP	JWGT
Volvox aureus	Transcriptome, 1KP	WRSL
Volvox globator	Transcriptome, 1KP	ISPU
**Pedinophyceae**		
Pedinomonas minor	Transcriptome, 1KP	RRSV
Pedinomonas tuberculata	Transcriptome, 1KP	PUAN
**Red Algae**		
Betaphycus gelatinae	Transcriptome, 1KP	BWVJ
Ceramium kondoi	Transcriptome, 1KP	VZWX
Chondrus crispus	Genome, Collen et al.[8]	-
Chondrus crispus	Transcriptome, 1KP	UGPM
Chroodactylon ornatum	Transcriptome, 1KP	LLXJ
Cyanidioschyzon merolae	Genome, Matsuzaki et al.[9]	-
Dumontia simplex	Transcriptome, 1KP	IEHF
Eucheuma denticulatum	Transcriptome, 1KP	JEBK
Galdieria sulphuraria	Genome, Barbier et al.[10]	-
Glaucosphaera vacuolata	Transcriptome, 1KP	RSOF
Gloeopeltis furcata	Transcriptome, 1KP	SBLT
Gracilaria asiatica	Transcriptome, 1KP	VNAL
Gracilaria blodgettii	Transcriptome, 1KP	LJPN
Gracilaria chouae	Transcriptome, 1KP	FTRP
Gracilaria lemaneiformi	Transcriptome, 1KP	IKWM
Grateloupia filicina	Transcriptome, 1KP	ZJOJ
Grateloupia livida	Transcriptome, 1KP	IKIZ
Grateloupia turuturu	Transcriptome, 1KP	URSB
Grateloupia chiangii	Transcriptome, 1KP	PWKQ
Gymnogongrus ftabelliformis	Transcriptome, 1KP	CKXF
Heterosiphonia pulchra	Transcriptome, 1KP	YSBD
Kappaphycus alvarezii	Transcriptome, 1KP	IHJY
Mazzaella japonica	Transcriptome, 1KP	WEJN
Polysiphonia japonica	Transcriptome, 1KP	XAXW
Pyropia yezoensis	Genome, Nakamura et al.[11]	-
Porphyra yezoensis	Transcriptome, 1KP	ZULJ

Porphyridium cruentum	Transcriptome, 1KP	OBUY
Porphyridium purpureum	Genome, Bhattacharya et al.[12]	-
Porphyridium purpureum	Transcriptome, 1KP	PVGP
Rhodella violacea	Transcriptome, 1KP	RTLC
Rhodochaete parvula	Transcriptome, 1KP	JJZR
Sinotubimorpha guangdongensis	Transcriptome, 1KP	PYDB
Symphyocladia latiuscula	Transcriptome, 1KP	UYFR
**Prasinophyte**		
Monomastix opisthostigma	Transcriptome, 1KP	BTFM
Pyramimonas parkeae	Transcriptome, 1KP	TNAW
Tetraselmis cordiformis	Transcriptome, 1KP	DUMA
**Cryptophyte**		
Chroomonas sp	Transcriptome, 1KP	ROZZ
Cryptomonas curvata	Transcriptome, 1KP	BAKF
Guillardia theta	Genome, Curtis et al.[13]	-
Hemiselmis virescens	Transcriptome, 1KP	MJMQ
Rhodomonas sp	Transcriptome, 1KP	IAYV
**Glaucophyte**		
Cyanophora paradoxa	Genome, Price et al.[14]	-
Cyanoptyche gloeocystis	Transcriptome, 1KP	JKHA
Glaucocystis nostochinearum	Transcriptome, 1KP	POOW
**Haptophytes**		
Isochrysis sp.	Transcriptome, 1KP	BAJW
Pavlova lutheri	Transcriptome, 1KP	NMAK
Prymnesium parvum	Transcriptome, 1KP	LXRN
**Stramenopiles - Chrysophytes**		
Mallomonas sp.	Transcriptome, 1KP	BOGT
Ochromonas sp.	Transcriptome, 1KP	EBWI
Synura petersenii	Transcriptome, 1KP	DBYD
Synura sp.	Transcriptome, 1KP	VKVG
**Stramenopiles - Brown algae**		
Ishige okamurae	Transcriptome, 1KP	APTP
Petalonia fascia	Transcriptome, 1KP	VRGZ
Scytosiphon lomentaria	Transcriptome, 1KP	JCXF

Supplementary Table 2. GenBank accessions of the phytochromes used in phylogenetic analyses. "-": not applicable.

Phytochrome	Source	1KP[3] identififer	GenBank accession
**Angiosperm PHYA**			
Arabidopsis thaliana	Genome, Phytozome[2]	-	-
Fragaria vesca	Genome, Phytozome	-	-
Phaseolus vulgaris 1	Genome, Phytozome	-	-
Phaseolus vulgaris 2	Genome, Phytozome	-	-
Cucumis sativus 1	Genome, Phytozome	-	-
Cucumis sativus 2	Genome, Phytozome	-	-
Vitis vinifera	Genome, Phytozome	-	-
Solanum lycopersicum	Genome, Phytozome	-	-
Buxus sempervirens	Transcriptome, 1KP[2]	IWMW	KT071960
Aquilegia coerulea	Genome, Phytozome	-	-
Oryza sativa	Genome, Phytozome	-	-
Platanthera clavellata	Transcriptome, 1KP	MTHW	KT071961
Pistia stratioides	Transcriptome, 1KP	MFIN	KT071962
Laurelia sempervirens	Transcriptome, 1KP	WAIL	KT071963
Peperomia fraseri	Transcriptome, 1KP	XSZI	KT071964
Illicium floridanum	Transcriptome, 1KP	VZCI	KT071965
Amborella trichocarpa	Genome, Amborella Genome Project[1]	-	-
**Gymnosperm PHYN**			
Cephalotaxus harringtonia	Transcriptome, 1KP	WYAJ	KT071966
Podocarpus rubens	Transcriptome, 1KP	XLGK	KJ195161
Thuja plicata	Transcriptome, 1KP	VFYZ	KT071967
Taiwania cryptomerioides	Transcriptome, 1KP	QSNJ	KJ195162
Pinus parviflora	Transcriptome, 1KP	IIOL	KJ195163
Welwitschia mirabilis	Transcriptome, 1KP	TOXE	KJ195164
Gnetum montanum	Transcriptome, 1KP	GTHK	KJ195165
Ginkgo biloba	Transcriptome, 1KP	SGTW	KT071968
Encephalartos barteri	Transcriptome, 1KP	GNQG	KJ195166
**Angiosperm PHYC**			
Arabidopsis thaliana	Genome, Phytozome	-	-
Fragaria vesca	Genome, Phytozome	-	-
Cucumis sativus	Genome, Phytozome	-	-
Vitis vinifera	Genome, Phytozome	-	-
Solanum lycopersicum	Genome, Phytozome	-	-
Buxus sempervirens	Transcriptome, 1KP	IWMW	KT071969
Aquilegia coerulea	Genome, Phytozome	-	-
Oryza sativa	Genome, Phytozome	-	-
Platanthera clavellata	Transcriptome, 1KP	MTHW	KT071970
Pistia stratioides	Transcriptome, 1KP	MFIN	KT071971
Laurelia sempervirens	Transcriptome, 1KP	WAIL	KT071972
Peperomia fraseri	Transcriptome, 1KP	XSZI	KT071973
Illicium floridanum	Transcriptome, 1KP	VZCI	KT071974
Amborella trichocarpa	Genome, Amborella Genome Project	-	-
**Gymnosperm PHYO**			
Podocarpus rubens	Transcriptome, 1KP	XLGK	KJ195167
Thuja plicata	Transcriptome, 1KP	VFYZ	KT071975
Taiwania cryptomerioides	Transcriptome, 1KP	QSNJ	KJ195168
Pinus parviflora	Transcriptome, 1KP	IIOL	KJ195169
Ginkgo biloba	Transcriptome, 1KP	SGTW	KT071976
Encephalartos barteri	Transcriptome, 1KP	GNQG	KJ195170

**Angiosperm PHYB**			
Arabidopsis thaliana D	Genome, Phytozome	-	-
Arabidopsis thaliana B	Genome, Phytozome	-	-
Fragaria vesca	Genome, Phytozome	-	-
Phaseolus vulgaris	Genome, Phytozome	-	-
Cucumis sativus	Genome, Phytozome	-	-
Vitis vinifera	Genome, Phytozome	-	-
Solanum lycopersicum 1	Genome, Phytozome	-	-
Solanum lycopersicum 2	Genome, Phytozome	-	-
Buxus sempervirens	Transcriptome, 1KP	IWMW	KT071977
Aquilegia coerulea	Genome, Phytozome	-	-
Oryza sativa	Genome, Phytozome	-	-
Platanthera clavellata	Transcriptome, 1KP	MTHW	KT071978
Pistia stratioides	Transcriptome, 1KP	MFIN	KT071979
Laurelia sempervirens	Transcriptome, 1KP	WAIL	KT071980
Peperomia fraseri	Transcriptome, 1KP	XSZI	KT071981
Illicium floridanum	Transcriptome, 1KP	VZCI	KT071982
Amborella trichocarpa	Genome, Amborella Genome Project	-	-
**Angiosperm PHYE**			
Arabidopsis thaliana	Genome, Phytozome	-	-
Fragaria vesca 1	Genome, Phytozome	-	-
Fragaria vesca 2	Genome, Phytozome	-	-
Phaseolus vulgaris	Genome, Phytozome	-	-
Cucumis sativus	Genome, Phytozome	-	-
Solanum lycopersicum	Genome, Phytozome	-	-
Capnoides sempervirens 1	Transcriptome, 1KP	AUGV	KT071983
Aquilegia coerulea 1	Genome, Phytozome	-	-
Capnoides sempervirens 2	Transcriptome, 1KP	AUGV	KT071984
Aquilegia coerulea 2	Genome, Phytozome	-	-
Laurelia sempervirens	Transcriptome, 1KP	WAIL	KT071985
Illicium floridanum	Transcriptome, 1KP	VZCI	KT071986
**Gymnosperm PHYP**			
Thuja plicata	Transcriptome, 1KP	VFYZ	KT071987
Taiwania cryptomerioides	Transcriptome, 1KP	QSNJ	KJ195155
Cephalotaxus harringtonia	Transcriptome, 1KP	WYAJ	KT071988
Podocarpus rubens	Transcriptome, 1KP	XLGK	KJ195156
Pinus parviflora	Transcriptome, 1KP	IIOL	KJ195157
Gnetum montanum	Transcriptome, 1KP	GTHK	KJ195158
Welwitschia mirabilis	Transcriptome, 1KP	TOXE	KJ195159
Ginkgo biloba	Transcriptome, 1KP	SGTW	KT071989
Encephalartos barteri	Transcriptome, 1KP	GNQG	KJ195160
**Fern PHY1**			
Equisetum diffusum	Transcriptome, 1KP	CAPN	KT071819
Marattia howeana	Cloning, this study	-	KP872700
Osmunda sp	Transcriptome, 1KP	UOMY	KJ195150
Dipteris conjugata	Transcriptome, 1KP	MEKP	KT071820
Pilularia globulifera	Transcriptome, 1KP	KIIX	KJ195151
Plagiogyria japonica	Transcriptome, 1KP	UWOD	KJ195152
Adiantum capillus-veneris	GenBank	-	AB016151
Pteridium aquilinum	Transcriptome, Der et al.[5]	-	SRX423244
Cystopteris protrusa	Transcriptome, 1KP	YOWV	KT071821
Blechnum spicant	Transcriptome, 1KP	VITX	KJ195153
Polypodium glycyrrhiza	Transcriptome, 1KP	CJNT	KJ195154

Asplenium platyneuron	Transcriptome, 1KP	KJZG	KT071822
Alsophila spinulosa	Transcriptome, 1KP	GANB	KT071823
Woodsia scopulina	Transcriptome, 1KP	YJJY	KT071824
Deparia lobato-crenata	Transcriptome, 1KP	FCHS	KT071825
Vittaria appalachiana	Transcriptome, 1KP	NDUV	KT071826
Danaea nodosa	Transcriptome, 1KP	DFHO	KT071827
Azolla filiculoides	Transcriptome, Brouwer et al.[4]	-	-
Thyrsopteris elegans	Transcriptome, 1KP	EWXK	KT071828
Ceratopteris thalictroides	Transcriptome, 1KP	PIVW	KT071829
Lonchitis hirsuta	Transcriptome, 1KP	VVRN	KT071830
**Fern PHY2/4**			
Equisetum diffusum A	Transcriptome, 1KP	CAPN	KJ195136
Equisetum diffusum B	Transcriptome, 1KP	CAPN	KT071831
Botrypus virginianus A	Transcriptome, 1KP	BEGM	KJ195137
Sceptridium dissectum A	Transcriptome, 1KP	EEAQ	KT071832
Botrypus virginianus B	Transcriptome, 1KP	BEGM	KT071833
Sceptridium dissectum B	Transcriptome, 1KP	EEAQ	KT071834
Psilotum nudum	GenBank	QVMR	X74930
Marattia attenuata	Transcriptome, 1KP	UGNK	KT071835
Osmunda sp A	Transcriptome, 1KP	UOMY	KJ195138
Dipteris conjugata	Transcriptome, 1KP	MEKP	KJ195139
Danaea nodosa	Transcriptome, 1KP	DFHO	KT071836
Osmundastrum cinnamomeum A	Transcriptome, 1KP	RFMZ	KT071837
Osmunda javanica A	Transcriptome, 1KP	VIBO	KT071838
Osmundastrum cinnamomeum B	Transcriptome, 1KP	RFMZ	KT071839
Osmunda javanica B	Transcriptome, 1KP	VIBO	KT071840
**Fern PHY2**			
Anemia tomentosa	Transcriptome, 1KP	CQPW	KJ195140
Pilularia globulifera	Transcriptome, 1KP	KIIX	KJ195142
Plagiogyria japonica	Transcriptome, 1KP	UWOD	KJ195141
Adiantum capillus-veneris	GenBank	-	AB016232
Pteridium aquilinum	Transcriptome, Der et al.	-	SRX423244
Polystichum acrostichoides	Transcriptome, 1KP	FQGQ	KT071841
Cystopteris fragilis	Transcriptome, 1KP	LHLE	KT071842
Blechnum spicant	Transcriptome, 1KP	VITX	KJ195143
Leucostegia immersa	Transcriptome, 1KP	WGTU	KT071843
Polypodium hesperium	Transcriptome, 1KP	GYFU	KJ195144
Gymnocarpium dryopteris	Transcriptome, 1KP	HEGQ	KT071844
Homalosorus pycnocarpos	Transcriptome, 1KP	OCZL	KT071845
Athyrium filix-femina	Transcriptome, 1KP	AFPO	KT071846
Diplazium wichurae	Transcriptome, 1KP	UFJN	KT071847
Deparia lobato-crenata	Transcriptome, 1KP	FCHS	KT071848
Asplenium platyneuron	Transcriptome, 1KP	KJZG	KT071849
Woodsia scopulina	Transcriptome, 1KP	YJJY	KT071850
Woodsia ilvensis	Transcriptome, 1KP	YQEC	KT071851
Vittaria appalachiana	Transcriptome, 1KP	NDUV	KT071852
Pityrogramma trifoliata	Transcriptome, 1KP	UJTT	KT071853
Pteris ensiformis	Transcriptome, 1KP	FLTD	KT071854
Myriopteris rufa	Transcriptome, 1KP	GSXD	KT071855
Gaga arizonica	Transcriptome, 1KP	DCDT	KT071856
Argyrochosma nivea	Transcriptome, 1KP	XDDT	KT071857
Notholaena montieliae	Transcriptome, 1KP	YCKE	KT071858
Cryptogramma acrostichoides	Transcriptome, 1KP	WQML	KT071859

Thyrsopteris elegans	Transcriptome, 1KP	EWXK	KT071860
Culcita macrocarpa	Transcriptome, 1KP	PNZO	KT071861
Lindsaea microphylla	Transcriptome, 1KP	YIXP	KT071862
Lonchitis hirsuta	Transcriptome, 1KP	VVRN	KT071863
Ceratopteris thalictroides	Transcriptome, 1KP	PIVW	KT071864
**Fern PHY4A**			
Plagiogyria japonica	Transcriptome, 1KP	UWOD	KJ195145
Pilularia globulifera	Transcriptome, 1KP	KIIX	KJ195146
Adiantum tenerum	Transcriptome, 1KP	BMJR	KJ195147
Polystichum acrostichoides	Transcriptome, 1KP	FQGQ	KT071865
Cystopteris fragilis	Transcriptome, 1KP	LHLE	KT071866
Blechnum spicant	Transcriptome, 1KP	VITX	KJ195148
Leucostegia immersa	Transcriptome, 1KP	WGTU	KT071867
Polypodium hesperium	Transcriptome, 1KP	GYFU	KJ195149
Adiantum capillus-veneris	GenBank	-	AB003364
Asplenium platyneuron	Transcriptome, 1KP	KJZG	KT071868
Gaga arizonica	Transcriptome, 1KP	DCDT	KT071869
Myriopteris rufa	Transcriptome, 1KP	GSXD	KT071870
Argyrochosma nivea	Transcriptome, 1KP	XDDT	KT071871
Vittaria appalachiana	Transcriptome, 1KP	NDUV	KT071872
Pteris ensiformis	Transcriptome, 1KP	FLTD	KT071873
Cryptogramma acrostichoides	Transcriptome, 1KP	WQML	KT071874
Alsophila spinulosa	Transcriptome, 1KP	GANB	KT071875
Azolla caroliniana	Transcriptome, 1KP	CVEG	KT071876
Lonchitis hirsuta	Transcriptome, 1KP	VVRN	KT071877
Ceratopteris thalictroides	Transcriptome, 1KP	PIVW	KT071878
Anemia tomentosa A	Transcriptome, 1KP	CQPW	KT071879
Anemia tomentosa B	Transcriptome, 1KP	CQPW	KT071880
Lindsaea microphylla	Transcriptome, 1KP	YIXP	KT071881
Lygodium japonicum	Transcriptome, 1KP	PBUU	KT071882
Thyrsopteris elegans	Transcriptome, 1KP	EWXK	KT071883
**Fern PHY4B**			
Pteridium aquilinum	Transcriptome, Der et al.	-	SRX423244
Cystopteris fragilis	Transcriptome, 1KP	LHLE	KT071884
Blechnum spicant	Transcriptome, 1KP	VITX	KT071885
Polystichum acrostichoides	Transcriptome, 1KP	FQGQ	KT071886
Gymnocarpium dryopteris	Transcriptome, 1KP	HEGQ	KT071887
Woodsia ilvensis	Transcriptome, 1KP	YQEC	KT071888
Athyrium filix-femina	Transcriptome, 1KP	AFPO	KT071889
Diplazium wichurae	Transcriptome, 1KP	UFJN	KT071890
**Isoetopsida PHY**			
Selaginella cf pallescens	Transcriptome, 1KP	ABIJ	KT071891
Selaginella moellendorffii	Genome, Phytozome	-	XM_002991595
Selaginella willdenowii	Transcriptome, 1KP	KJYC	KT071892
Selaginella kraussiana	Transcriptome, 1KP	ZFGK	KT071893
Selaginella wallacei	Transcriptome, 1KP	JKAA	KJ195172
Selaginella apoda	Transcriptome, 1KP	LGDQ	KT071894
Isoetes tegetiformans	Transcriptome, 1KP	PKOX	KJ195171
**Lycopodiales PHY1**			
Dendrolycopodium obscurum	Transcriptome, 1KP	XNXF	KJ195173
Huperzia selago	Transcriptome, 1KP	GTUO	KJ195174
Pseudolycopodiella caroliniana	Transcriptome, 1KP	UPMJ	KT071895
**Lycopodiales PHY2**			

Dendrolycopodium obscurum	Transcriptome, 1KP	XNXF	KJ195175
Huperzia lucidula	Transcriptome, 1KP	GKAG	KJ195176
Pseudolycopodiella caroliniana	Transcriptome, 1KP	UPMJ	KT071896
**Moss PHY1_3**			
Andreaea rupestris	Transcriptome, 1KP	WOGB	KT071897
Rhynchostegium serrulatum	Transcriptome, 1KP	JADL	KT071898
Aulacomnium heterostichum	Transcriptome, 1KP	WNGH	KT071899
Hedwigia ciliata	Transcriptome, 1KP	YWNF	KJ195194
Philonotis fontana	Transcriptome, 1KP	ORKS	KT071900
Ceratodon purpureus A	GenBank	-	U72993
Ceratodon purpureus B	GenBank	-	U87632
Leucobryum albidum	Transcriptome, 1KP	VMXJ	KT071901
Physcomitrella patens 1	Genome, Phytozome	-	XM_001765983
Physcomitrella patens 3	Genome, Phytozome	-	XM_001778103
Racomitrium varium	Transcriptome, 1KP	RDOO	KT071902
Leucodon julaceus	Transcriptome, 1KP	IGUH	KT071903
Leucodon brachypus	Transcriptome, 1KP	ZACW	KT071904
Pseudotaxiphyllum elegans	Transcriptome, 1KP	QKQO	KT071905
Neckera douglasii	Transcriptome, 1KP	TMAJ	KT071906
Stereodon subimponens	Transcriptome, 1KP	LNSF	KT071907
Loeskeobryum brevirostre	Transcriptome, 1KP	WSPM	KT071908
Cladopodium rostratum	Transcriptome, 1KP	VBMM	KJ195193
Bryum argenteum	Transcriptome, 1KP	JMXW	KJ195195
**Moss PHY2_4/5**			
Andreaea rupestris	Transcriptome, 1KP	WOGB	KT071909
Sphagnum palustre	Transcriptome, 1KP	RCBT	KT071910
Takakia lepidozioides	Transcriptome, 1KP	SKQD	KT071911
**Moss PHY2_4**			
Atrichum angustatum	Transcriptome, 1KP	ZTHV	KT071912
Physcomitrella patens 4	Genome, Phytozome	-	XM_001773498
Physcomitrella patens 2	Genome, Phytozome	-	XM_001782287
Rhynchostegium serrulatum	Transcriptome, 1KP	JADL	KT071913
Aulacomnium heterostichum	Transcriptome, 1KP	WNGH	KT071914
Hedwigia ciliata	Transcriptome, 1KP	YWNF	KJ195190
Philonotis fontana	Transcriptome, 1KP	ORKS	KT071915
Ceratodon purpureus	Transcriptome, 1KP	FFPD	KT071916
Leucobryum albidum	Transcriptome, 1KP	VMXJ	KT071917
Racomitrium varium	Transcriptome, 1KP	RDOO	KT071918
Scouleria aquatica	Transcriptome, 1KP	BPSG	KT071919
Bryum argenteum	Transcriptome, 1KP	JMXW	KJ195191
Loeskeobryum brevirostre	Transcriptome, 1KP	WSPM	KT071920
Neckera douglasii	Transcriptome, 1KP	TMAJ	KT071921
Cladopodium rostratum	Transcriptome, 1KP	VBMM	KJ195189
Leucodon brachypus	Transcriptome, 1KP	ZACW	KT071922
Leucodon julaceus	Transcriptome, 1KP	IGUH	KT071923
Pseudotaxiphyllum elegans	Transcriptome, 1KP	QKQO	KT071924
Anomodon attenuatus	Transcriptome, 1KP	QMWB	KT071925
Stereodon subimponens	Transcriptome, 1KP	LNSF	KT071926
**Moss PHY5**			
Atrichum angustatum	Transcriptome, 1KP	ZTHV	KT071927
Physcomitrella patens A	Genome, Phytozome	-	XM_001761093
Physcomitrella patens B	Genome, Phytozome	-	XM_001767172
Physcomitrella patens C	Genome, Phytozome	-	XM_001754314

Rhynchostegium serrulatum D	Transcriptome, 1KP	JADL	KT071928
Aulacomnium heterostichum D	Transcriptome, 1KP	WNGH	KT071929
Hedwigia ciliata D	Transcriptome, 1KP	YWNF	KT071930
Philonotis fontana D	Transcriptome, 1KP	ORKS	KT071931
Leucobryum albidum D	Transcriptome, 1KP	VMXJ	KT071932
Ceratodon purpureus D	GenBank	-	AY123149
Scouleria aquatica D	Transcriptome, 1KP	BPSG	KT071933
Racomitrium varium D	Transcriptome, 1KP	RDOO	KT071934
Syntrichia princeps D	Transcriptome, 1KP	GRKU	KT071935
Bryum argenteum D	Transcriptome, 1KP	JMXW	KJ195187
Leucodon brachypus D	Transcriptome, 1KP	ZACW	KT071936
Anomodon attenuatus D	Transcriptome, 1KP	QMWB	KT071937
Cladopodium rostratum D	Transcriptome, 1KP	VBMM	KJ195185
Loeskeobryum brevirostre D	Transcriptome, 1KP	WSPM	KT071938
Pseudotaxiphyllum elegans D	Transcriptome, 1KP	QKQO	KT071939
Racomitrium varium E	Transcriptome, 1KP	RDOO	KT071940
Scouleria aquatica E	Transcriptome, 1KP	BPSG	KT071941
Syntrichia princeps E	Transcriptome, 1KP	GRKU	KT071942
Hedwigia ciliata E	Transcriptome, 1KP	YWNF	KJ195186
Philonotis fontana E	Transcriptome, 1KP	ORKS	KT071943
Leucobryum albidum E	Transcriptome, 1KP	VMXJ	KT071944
Ceratodon purpureus E	GenBank	-	EU122393
Rhynchostegium serrulatum E	Transcriptome, 1KP	JADL	KT071945
Aulacomnium heterostichum E 1	Transcriptome, 1KP	WNGH	KT071946
Aulacomnium heterostichum E 2	Transcriptome, 1KP	WNGH	KT071947
Anomodon attenuatus E	Transcriptome, 1KP	QMWB	KT071948
Neckera douglasii E	Transcriptome, 1KP	TMAJ	KT071949
Pseudotaxiphyllum elegans E	Transcriptome, 1KP	QKQO	KT071950
Cladopodium rostratum E	Transcriptome, 1KP	VBMM	KJ195184
Loeskeobryum brevirostre E	Transcriptome, 1KP	WSPM	KT071951
Leucodon julaceus E	Transcriptome, 1KP	IGUH	KT071952
Leucodon brachypus E	Transcriptome, 1KP	ZACW	KT071953
**Liverwort PHY**			
Scapania nemorosa	Transcriptome, 1KP	IRBN	KJ195177
Porella pinnata	Transcriptome, 1KP	UUHD	KJ195178
Schistochila sp	Transcriptome, 1KP	LGOW	KJ195179
Metzgeria crassipilis	Transcriptome, 1KP	NRWZ	KJ195180
Pellia neesiana	Transcriptome, 1KP	JHFI	KJ195181
Odontoschisma prostratum	Transcriptome, 1KP	YBQN	KT071954
Marchantia paleacea	GenBank	-	AB022917
Conocephalum conicum	Transcriptome, 1KP	ILBQ	KJ195182
Lunularia cruciata	Transcriptome, 1KP	TXVB	KT071955
Sphaerocarpos texanus	Transcriptome, 1KP	HERT	KJ195183
Ricciocarpos natans	Transcriptome, 1KP	WJLO	KT071956
Bazzania trilobata	Transcriptome, 1KP	WZYK	KT071957
**Hornwort PHY**			
Nothoceros aenigmaticus	Cloning, Li et al.[6]	DXOU	KJ128384
Megaceros flagellaris	Transcriptome, 1KP	UCRN	KJ195197
Paraphymatoceros hallii	Transcriptome, 1KP	FAJB	KJ195198
Phaeoceros carolinianus	Transcriptome, 1KP	WCZB	KJ195199
Anthoceros punctatus	Genome, Li et al.[6]	-	KJ195038
Phaeomegaceros coriaceus	Transcriptome, 1KP	AKXB	KT071958
Leiosporoceros dussii	Transcriptome, 1KP	ANON	KT071959

**Hornwort NEO**			
Nothoceros aenigmaticus	Transcriptome, 1KP	DXOU	KJ128382
Megaceros flagellaris	Transcriptome, 1KP	UCRN	KJ195132
Paraphymatoceros hallii	Transcriptome, 1KP	FAJB	KJ194999
Phaeoceros carolinianus	Transcriptome, 1KP	WCZB	KJ194998
Anthoceros punctatus	Cloning, Li et al.	-	KJ194997
**Fern NEO**			
Adiantum capillus-veneris	GenBank	-	AB012082
Dipteris conjugata	Cloning, Li et al.	-	KJ195011
Blechnum spicant	Cloning, Li et al.	-	KJ195007
Hemidictyum marginatum	Cloning, Li et al.	-	KJ195037
**Zygnematales NEO**			
Mougeotia scalaris 1	GenBank	-	AB206961
Mougeotia scalaris 2	GenBank	-	AB206962
Cylindrocystis brebissonii 1	Transcriptome, 1KP	YOXI	KJ195042
Cylindrocystis sp 1	Transcriptome, 1KP	VAZE	KJ195043
Cylindrocystis brebissonii 2	Transcriptome, 1KP	YOXI	KJ195044
Cylindrocystis sp 2	Transcriptome, 1KP	VAZE	KJ195041
Zygnemopsis sp	Transcriptome, 1KP	MFZO	KJ195039
**Desmidiales PHY2C**			
Desmidium aptogonum	Transcriptome, 1KP	DFDS	KT071990
Onychonema laeve	Transcriptome, 1KP	GGWH	KT071991
Cosmarium granatum 1	Transcriptome, 1KP	MNNM	KT071992
Staurodesmus convergens 1	Transcriptome, 1KP	WCQU	KT071993
Cosmarium subtumidum	Transcriptome, 1KP	WDGV	KT071994
Staurodesmus convergens 2	Transcriptome, 1KP	WCQU	KT071995
Cosmarium granatum 2	Transcriptome, 1KP	MNNM	KT071996
Phymatodocis nordstedtiana	Transcriptome, 1KP	RPQV	KT071997
Penium exiguum	Transcriptome, 1KP	YSQT	KT071998
Gonatozygon kinahanii 1	Transcriptome, 1KP	KEYW	KT071999
Gonatozygon kinahanii 2	Transcriptome, 1KP	KEYW	KT072000
**Desmidiales PHY2B**			
Desmidium aptogonum	Transcriptome, 1KP	DFDS	KT072001
Onychonema laeve	Transcriptome, 1KP	GGWH	KT072002
Cosmarium subtumidum	Transcriptome, 1KP	WDGV	KT072003
Staurodesmus convergens	Transcriptome, 1KP	WCQU	KT072004
Phymatodocis nordstedtiana	Transcriptome, 1KP	RPQV	KT072005
Penium exiguum	Transcriptome, 1KP	YSQT	KT072006
Gonatozygon kinahanii	Transcriptome, 1KP	KEYW	KT072007
**Desmidiales PHY2A**			
Desmidium aptogonum	Transcriptome, 1KP	DFDS	KT072008
Cosmarium subtumidum	Transcriptome, 1KP	WDGV	KT072009
Cosmarium granatum	Transcriptome, 1KP	MNNM	KT072010
Onychonema laeve	Transcriptome, 1KP	GGWH	KT072011
Staurodesmus convergens	Transcriptome, 1KP	WCQU	KT072012
Phymatodocis nordstedtiana	Transcriptome, 1KP	RPQV	KT072013
Penium exiguum	Transcriptome, 1KP	YSQT	KT072014
Gonatozygon kinahanii	Transcriptome, 1KP	KEYW	KT072015
Roya obtusa	Transcriptome, 1KP	XRTZ	KJ195200
Planotaenium ohtanii	Transcriptome, 1KP	SNOX	KJ195201
**Zygnematales PHY2**			
Mougeotia scalaris	Genbank	-	AB206965
Mesotaenium kramstae	Transcriptome, 1KP	NBYP	KT072016

Mesotaenium caldariorum	Genbank	-	U31283
Zygnemopsis sp	Transcriptome, 1KP	MFZO	KJ195202
Cylindrocystis sp	Transcriptome, 1KP	VAZE	KJ195203
Cylindrocystis brebissonii	Transcriptome, 1KP	YOXI	KJ195204
Mesotaenium braunii	Transcriptome, 1KP	WSJO	KJ195205
Netrium digitus	Transcriptome, 1KP	FFGR	KT072017
**Coleochaetales PHY2**			
Coleochaete irregularis	Transcriptome, 1KP	QPDY	KJ195206
Chaetosphaeridium globosum	Transcriptome, 1KP	DRGY	KJ195207
**Klebsormidiales PHY2**			
Interfilum paradoxum	Transcriptome, 1KP	FPCO	KJ195208
Klebsormidium subtile	Transcriptome, 1KP	FQLP	KJ195209
Entransia fimbriata	Transcriptome, 1KP	BFIK	KJ195210
**Desmidiales PHY1**			
Cosmarium granatum	Transcriptome, 1KP	MNNM	KT072018
Cosmarium subtumidum	Transcriptome, 1KP	WDGV	KT072019
Staurodesmus convergens	Transcriptome, 1KP	WCQU	KT072020
Onychonema laeve	Transcriptome, 1KP	GGWH	KT072021
Phymatodocis nordstedtiana	Transcriptome, 1KP	RPQV	KT072022
Penium exiguum	Transcriptome, 1KP	YSQT	KT072023
Gonatozygon kinahanii	Transcriptome, 1KP	KEYW	KT072024
Roya obtusa	Transcriptome, 1KP	XRTZ	KT072025
Planotaenium ohtanii	Transcriptome, 1KP	SNOX	KT072026
**Klebsormidiales PHY1**			
Entransia fimbriata	Transcriptome, 1KP	BFIK	KT072027
**Mesostigmatales PHY1/2**			
Mesostigma viride	Transcriptome, 1KP	KYIO	KT072028
Chlorokybus atmophyticus 1	Transcriptome, 1KP	AZZW	KT072029
Chlorokybus atmophyticus 2	Transcriptome, 1KP	AZZW	KT072030
Spirotaenia minuta	Transcriptome, 1KP	NNHQ	KT072031
**Zygnematales PHYX2**			
Zygnemopsis sp	Transcriptome, 1KP	MFZO	KT072032
Cylindrocystis cushleckae	Transcriptome, 1KP	JOJQ	KT072033
Mesotaenium kramstae	Transcriptome, 1KP	NBYP	KT072034
Netrium digitus	Transcriptome, 1KP	FFGR	KT072035
**Coleochaetales PHYX2**			
Coleochaete irregularis	Transcriptome, 1KP	QPDY	KT072036
Chaetosphaeridium globosum	Transcriptome, 1KP	DRGY	KT072037
**Zygnematales PHYX1**			
Zygnemopsis sp	Transcriptome, 1KP	MFZO	KT072038
Mesotaenium kramstae	Transcriptome, 1KP	NBYP	KT072039
Cylindrocystis cushleckae	Transcriptome, 1KP	JOJQ	KT072040
Cylindrocystis brebissonii	Transcriptome, 1KP	YOXI	KT072041
Cylindrocystis sp	Transcriptome, 1KP	VAZE	KT072042
Netrium digitus	Transcriptome, 1KP	FFGR	KT072043
**Coleochaetales PHYX1**			
Coleochaete irregularis	Transcriptome, 1KP	QPDY	KT072044
**Prasinophyte PHY**			
Monomastix opisthostigma	Transcriptome, 1KP	BTFM	-
Pyramimonas parkeae	Transcriptome, 1KP	TNAW	-
Tetraselmis cordiformis	Transcriptome, 1KP	DUMA	-
Dolichomastix tenuilepsis	GenBank	-	KF876183
Tetraselmis astigmatica	GenBank	-	KF876182

Prasinoderma coloniale	GenBank	-	KF876180
Nephroselmis pyriformis	GenBank	-	KF876181
Micromonas pusilla	GenBank	-	KF754357
**Cryptophyte PHY**			
Hemiselmis virescens	Transcriptome, 1KP	MJMQ	KT013280
Cryptomonas curvata	Transcriptome, 1KP	BAKF	KT013281
Rhodomonas sp	Transcriptome, 1KP	IAYV	KT013282
Guillardia theta 1	Genome, Curtis et al.[13]	-	-
Guillardia theta 2	Genome, Curtis et al.	-	-
Chroomonas sp	Transcriptome, 1KP	ROZZ	KT013283
Guillardia theta PEK1	Genome, Curtis et al.	-	-
Guillardia theta PEK2	Genome, Curtis et al.	-	-
Guillardia theta PEK3	Genome, Curtis et al.	-	-
**Glaucophyte PHY**			
Cyanophora paradoxa 1	Genome, Price et al.[14]	-	KF597305
Cyanophora paradoxa 2	Genome, Price et al.	-	KF615870
Cyanophora paradoxa 4	Genome, Price et al.	-	-
Gloeochaete wittrockiana GPS2	Transcriptome, Duanmu et al.[15]	-	-
Gloeochaete wittrockiana GPS4	Transcriptome, Duanmu et al.	-	-
Gloeochaete wittrockiana GPS3	Transcriptome, Duanmu et al.	-	-
Gloeochaete wittrockiana GPS1	Transcriptome, Rockwell et al.[16]	-	KF894953
Cyanoptyche gloeocystis 1	Transcriptome, 1KP	JKHA	KT013284
Cyanoptyche gloeocystis 2	Transcriptome, 1KP	JKHA	KT013285
Cyanoptyche gloeocystis 3	Transcriptome, 1KP	JKHA	KT013286
Glaucocystis nostochinearum 1	Transcriptome, 1KP	POOW	KT013287
Glaucocystis nostochinearum 2	Transcriptome, 1KP	POOW	KT013288
Glaucocystis nostochinearum 3	Transcriptome, 1KP	POOW	KT013289
Glaucocystis nostochinearum 4	Transcriptome, 1KP	POOW	KT013290
**Diatom PHY**			
Thalassiosira pseudonana	GenBank	-	XM_002290775
Phaeodactylum tricornutum	GenBank	-	XM_002179026
**Brown algae PHY**			
Petalonia fascia	Transcriptome, 1KP	VRGZ	KT003814
Scytosiphon lomentaria	Transcriptome, 1KP	JCXF	KT003815
Ishige okamurai	Transcriptome, 1KP	APTP	KT003816
Ectocarpus siliculosus	GenBank	-	FN649030
Feldmannia species virus isolate FsV	GenBank	-	EU916176
Ectocarpus siliculosus virus isolate EsV	GenBank	-	AF204951
**Fungi PHY**			
Aspergillus fumigatus	GenBank	-	DS499603
Ustilago maydis	GenBank	-	AACP01000210
Cochliobolus heterostrophus	GenBank	-	AY456024
Neurospora crassa 1	GenBank	-	BK004087
Neurospora crassa 2	GenBank	-	DQ128077
Penicillium marneffei	GenBank	-	XM_002144684
**Cyanobacteria PHY**			
Microcystis aeruginosa	GenBank	-	AP009552
Nodularia spumigena	GenBank	-	AAVW01000039
Synechocystis sp	GenBank	-	BA000022
Anabaena variabilis	GenBank	-	NC_007413
Cyanothece sp	GenBank	-	ABVE01000001
Nostoc sp	GenBank	-	BA000019
Tolypothrix sp 1	GenBank	-	AF309559

Tolypothrix sp 2	GenBank	-	AF309560
**Cyanobacteria PAS-less PHY**			
Microcoleus chthonoplastes	GenBank	-	DS989848
Lyngbya sp	GenBank	-	AAVU01000002
Nostoc punctiforme	GenBank	-	CP001037
Anabaena variabilis	GenBank	-	CP000117
Synechococcus sp	GenBank	-	ABD00881
**Bacteria PHY**			
Agrobacterium tumefaciens	GenBank	-	C58AE007869
Pseudomonas aeruginosa	GenBank	-	CH482383
Pseudomonas syringae	GenBank	-	AE016853
Ralstonia pickettii	GenBank	-	CP001068
Stenotrophomonas maltophilia	GenBank	-	AM743169
Rhodopseudomonas palustris	GenBank	-	BX572602
Oceanicola granulosus	GenBank	-	AAOT01000028
Methylocella silvestris	GenBank	-	CP001280
Sclerotinia sclerotiorum	GenBank	-	CH476645
Magnetospirillum magnetotacticum	GenBank	-	AAAP01003476

Supplementary references

[1]Amborella Genome Project. The *Amborella* genome and the evolution of flowering plants. *Science* **342,** 1241089 (2013).

[2]Goodstein, D. M. *et al.* Phytozome: a comparative platform for green plant genomics. *Nucleic Acids Res.* **40,** D1178–86 (2012).

[3]Matasci, N. *et al*. Data access for the 1,000 Plants (1KP) project. *GigaScience* **3**, 17 (2014).

[4]Brouwer, P. *et al. Azolla* domestication towards a biobased economy? *New Phytol* **202,** 1069–1082 (2014).

[5]Der, J. P., Barker, M. S., Wickett, N. J., Depamphilis, C. W. & Wolf P. G. De novo characterization of the gametophyte transcriptome in bracken fern, *Pteridium aquilinum*. *BMC Genomics* **12,** 99 (2011).

[6]Li, F.-W. *et al*. Horizontal transfer of an adaptive chimeric photoreceptor from bryophytes to ferns. *Proc. Natl. Acad. Sci. USA* **111,** 6672–6677 (2014).

[7]Blanc, G. *et al.* The *Chlorella variabilis* NC64A genome reveals adaptation to photosymbiosis, coevolution with viruses, and cryptic sex. *Plant Cell* **22,** 2943–2955 (2010).

[8]Collen, J. *et al*. Genome structure and metabolic features in the red seaweed *Chondrus crispus* shed light on evolution of the Archaeplastida. *Proc. Natl. Acad. Sci. USA* **110,** 5247–5252 (2013).

[9]Matsuzaki, M. *et al*. Genome sequence of the ultrasmall unicellular red alga *Cyanidioschyzon merolae* 10D. *Nature* **428,** 653–657 (2004).

[10]Barbier, G. *et al.* Comparative genomics of two closely related unicellular thermo-acidophilic red algae, *Galdieria sulphuraria* and *Cyanidioschyzon merolae*, reveals the molecular basis of the metabolic flexibility of Galdieria sulphuraria and significant differences in carbohydrate metabolism of both algae. *Plant Physiology* **137,** 460–474 (2005).

[11]Nakamura, Y. *et al*. The first symbiont-free genome sequence of marine red alga, Susabi-nori (*Pyropia yezoensis*). *PLoS ONE* **8**, e57122 (2013).

[12]Bhattacharya, D. *et al*. Genome of the red alga *Porphyridium purpureum*. *Nat Comms* **4**, 1941 (2013).

[13]Curtis, B. A. *et al.* Algal genomes reveal evolutionary mosaicism and the fate of nucleomorphs. *Nature* **492**, 59–65 (2012).

[14]Price, D. C. *et al. Cyanophora paradoxa g*enome elucidates origin of photosynthesis in algae and plants. *Science* **335,** 843–847 (2012).

[15]Duanmu, D. *et al.* Marine algae and land plants share conserved phytochrome signaling systems. *Proc. Natl. Acad. Sci. USA* **111,** 15827–15832 (2014).

[16]Rockwell, N. C. *et al.* Eukaryotic algal phytochromes span the visible spectrum. *Proc. Natl. Acad. Sci. USA* **111,** 3871–3876 (2014).
